# Supplementary material for: TIP30 counteracts cardiac hypertrophy and failure by inhibiting translational elongation
Source: EMBO Mol Med. 2019 Aug 30;11(10):e10018. doi: 10.15252/emmm.201810018 (PMC6783653; doi:10.15252/emmm.201810018)
Supplement: Supplementary file 1 — Appendix [file EMMM-11-e10018-s001.pdf]

APPENDIX

Appendix Figure S1

Appendix Figure S2

Appendix Figure S3

Appendix Figure Legends

Appendix Table S1

Appendix Table S2

Appendix Table S3

Appendix Table S4

Appendix Table S5

Appendix Table S6

Appendix Figure S1

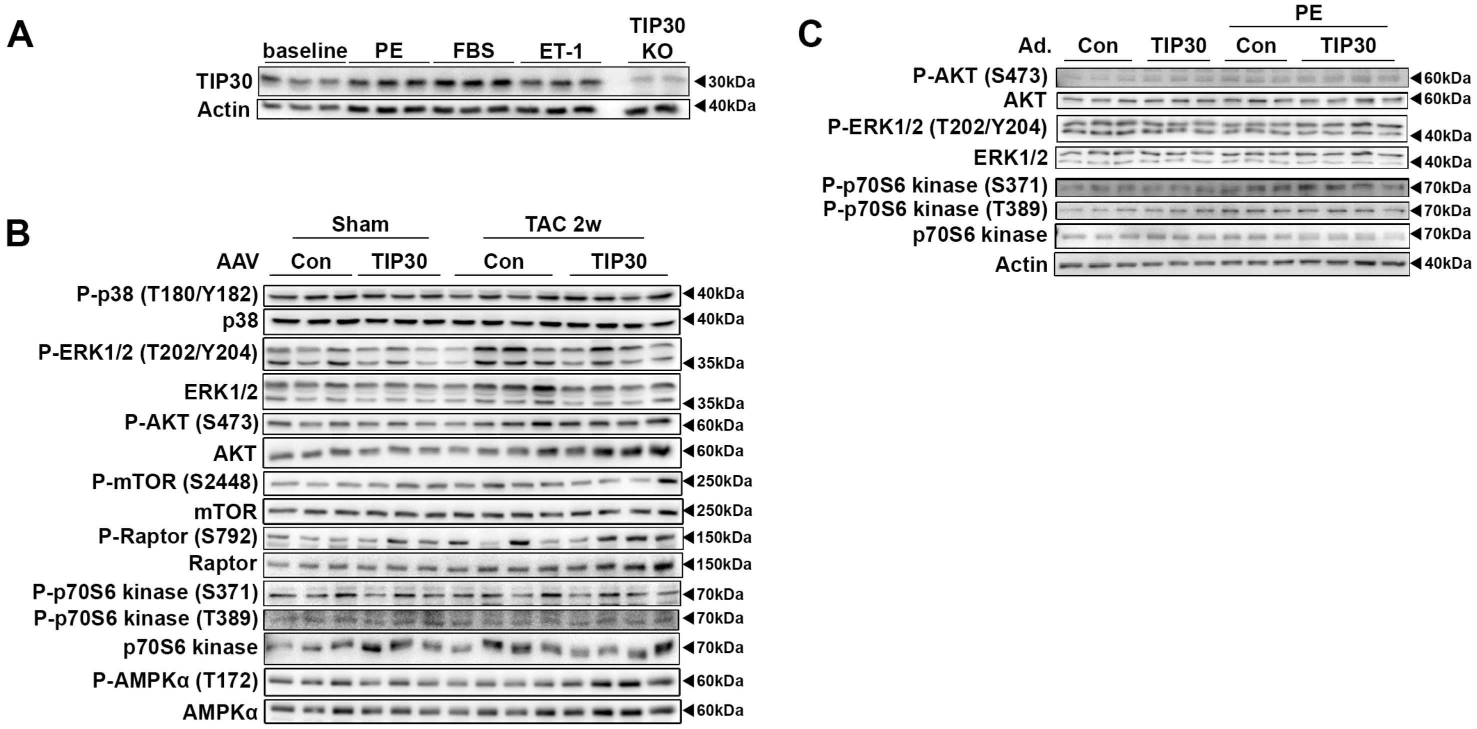

Appendix Figure S2

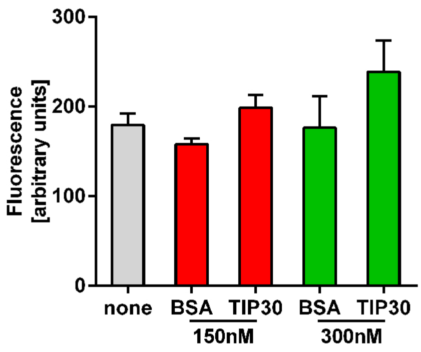

Appendix Figure S3

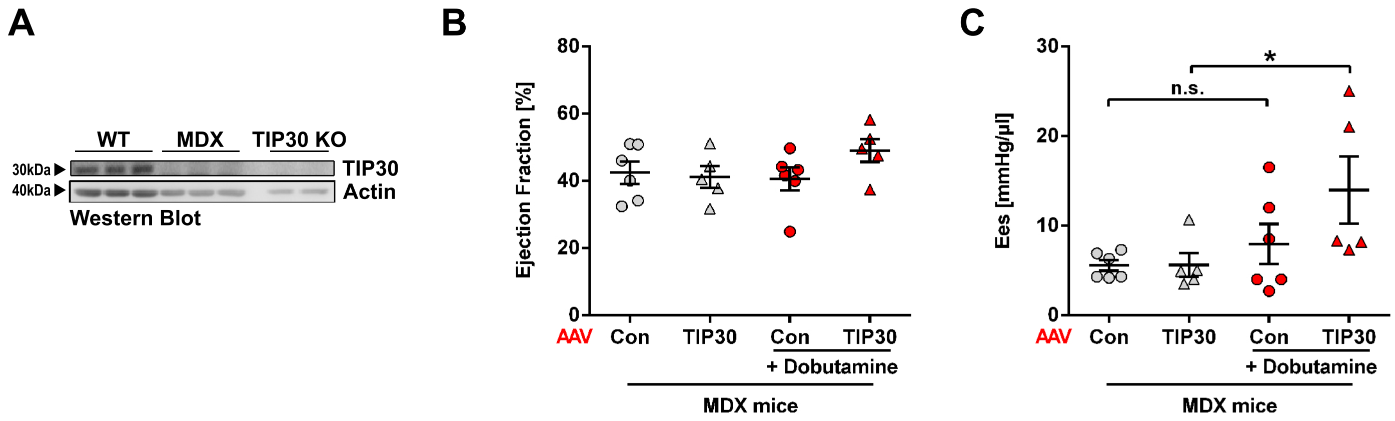

Appendix Figure Legends

Appendix Figure S1: Effects of TIP30 overexpression on hypertrophic signalling.

(A) Western Blot analysis of TIP30 and Actin from NRCM stimulated either with PE, FBS or endothelin-1 (ET-1).

(B) Western Blot analysis of indicated proteins in hearts from C57BL/6 WT mice treated either with AAV9 control virus (AAV-Con) or AAV9-TIP30 followed by 2 weeks TAC or sham surgery.

(C) Western Blot analysis of indicated proteins in neonatal rat cardiomyocytes (NRCM) after adenoviral transduction with control virus (Ad.Con) or Ad.TIP30 and stimulation with phenylephrine (PE) as indicated.

Appendix Figure S2: TIP30 does not interfere with tRNA binding by eEF1A1.

Quantification of fluorescent tRNA bound to eEF1A1 after incubation with purified TIP30-His or bovine serum albumin (BSA) as control (N=2 replicates/group).

Appendix Figure S3: Expression and functional effects of TIP30 in a mouse model of genetic cardiomyopathy.

(A) Western Blot analysis of TIP30 and Actin expression in WT or mdx (MDX) mice, TIP30 homozygous knock-out mice (KO) served as control.

(B; C) Quantification of ejection fraction (**B**, N=5 to 6 hearts/group) and endsystolic elastance (Ees; **C**, N=5 to 6 hearts/group) by left ventricular catheterization in 6mo old MDX mice with and without AAV9-mediated overexpression of TIP30 and Dobutamine injection as indicated. (n.s.): not statistically significant. *P<0.05. One-way ANOVA with Sidak´s multiple comparisons test.

Appendix Table S1: Candidate proteins co-precipitated with GST-TIP30 from cardiomyocyte lysate

**Protein**	**No. of peptides**	**Coverage (%)**	**Uniprot Accession Number**
Fructose-bisphosphate aldolase A	19	45.9	**P05065**
Polymerase I and transcript release factor **Ptrf**	13	43.9	**P85125**
40S ribosomal protein S3a **Rps3a**	15	37.5	**P49242**
Trifunctional enzyme subunit alpha, mitochondrial	44	37.1	**Q64428**
Glyceraldehyde-3-phosphate dehydrogenase **GAPDH**	18	35.8	**P04797**
ADP/ATP translocase 2	14	31.5	**Q09073**
Nucleolin **Ncl**	9	30.9	**P13383**
Basic leucine zipper and W2 domain-containing protein 2 **Bzw2**	10	24.8	**Q9WTT7**
Elongation factor 1-alpha 1 **eEF1A1**	16	24.6	**P62630**
Heterogeneous nuclear ribonucleoproteins A2/B1 **hnRNP A2/B1**	5	15	**A7VJC2**
Succinyl-CoA ligase [GDP-forming] subunit alpha, mitochondrial	6	10.3	**P13086**
Cytoplasmic dynein 1 heavy chain 1	16	7.2	**P38650**

Appendix Table S2: Clinical data of the HCM patients

Sex	Diagnosis	Age at operation [years]	IVS [mm]	known mutations
f	HOCM	62	20	negative in MYH7, MYBPC3, TNNT2
f	HOCM	19	33	MYH7, TNNT2
m	HOCM	67	14	negative in MYH7, MYBPC3, TNNT2
m	HOCM	22	50	negative in MYH7, MYBPC3, TNNT2
f	HOCM	15	>>13	MYBPC3
m	FHC	55	n/a	MYH7
m	HOCM	45	n/a	MYBPC3
m	HOCM	32	19 (at age 30)	MYH7

IVS: intraventricular septum; m: male; f: female; HOCM: hypertrophic obstructive cardiomyopathy; FHC: familial hypertrophic cardiomyopathy.

Appendix Table S3: Cardiomyocyte proteins regulated following adenoviral TIP30 overexpression as identified by DIGE

Protein	Regulation	Average Ratio	No. of peptides	Coverage (%)	Uniprot Accession Number
Receptor of activated protein C kinase 1 **Rack1**	Down	-1.28	5	19.2	P63245
Transitional endoplasmic reticulum ATPase **VCP**	Down	-1.24	12	17.4	P46462
Vinculin **Vcl**	Up	1.34	14	16.2	P85972
Chloride intracellular channel protein 5 **Clic5**	Up	1.36	4	13.9	Q9EPT8
Receptor of activated protein C kinase 1 **Rack1**	Up	1.41	5	20.2	P63245
ATP synthase subunit alpha, mitochondrial **Atp5a1**	Up	1.46	4	9.95	P15999
Chloride intracellular channel protein 5 **Clic5**	Up	2.05	2	9.56	Q9EPT8
NAD-dependent deacetylase sirtuin-5 **Sirt5**	Up	2.45	4	13.5	Q68FX9
Voltage-dependent anion-selective channel protein 3 **Vdac3**	Up	2.45	6	23.3	Q9R1Z0
Retinol dehydrogenase 14 **Rdh14**	Up	2.61	2	7.49	Q9ERI6
ATP synthase subunit alpha, mitochondrial **Atp5a1**	Up	2.82	6	14.3	P15999
Omega-amidase NIT2 **Nit2**	Up	2.98	8	33.7	Q497B0
ATP synthase subunit alpha, mitochondrial **Atp5a1**	Up	3.48	5	12.3	P15999
ATP synthase subunit alpha, mitochondrial **Atp5a1**	Up	4.89	7	15.4	P15999
ATP synthase subunit alpha, mitochondrial **Atp5a1**	Up	4.96	9	19.9	P15999
Malate dehydrogenase, mitochondrial **Mdh2**	Up	4.96	7	25.7	P04636
MICOS complex subunit Mic19 **Chchd3**	Up	5.66	2	5.3	Q9CRB9
ATP synthase subunit alpha, mitochondrial **Atp5a1**	Up	5.97	2	4.9	P15999
Oxidoreductase HTATIP2 **Tip30**	Up	100.80	4	19.4	Q9Z2G9

Appendix Table S4: Reagents, chemicals, plasmids, kits and mice used in this study

Reagent or Resource	SOURCE	IDENTIFIER	
**Antibodies**			**dilution**
anti-Actin	Sigma Aldrich	A2066	WB: 1:1.000
anti-Akt	Cell Signaling	9272	WB: 1:1.000
anti-AMPK-alpha	Cell Signaling	2532	WB: 1:1.000
Anti-cleaved caspase-3	Cell Signaling	9661	WB: 1:1.000
anti-eEF1A1	Abcam	118703	WB: 1:1.000; IF 1:100
Anti-eEF1A1+eEF1A2+eEF1AL3	Abcam	37969	WB: 1:1.000
Anti-eEF1A1	Sigma Aldrich	sc-21758	WB: 1:1.000
anti-eEF1A2	Abcam	156954	WB: 1:1.000
anti-eEF1B2	Abcam	77043	WB: 1:1.000
anti-GAPDH	Fitzgerald	10R-G109a	WB: 1:3.500
anti-GST	Cell Signaling	2625	WB: 1:1.000
anti-His	Cell Signaling	2365	WB: 1:1.000
anti-His	Cell Signaling	2366	WB: 1:1.000
anti-mTOR	Cell Signaling	2983	WB: 1:1.000
Anti-Myc	Cell Signalling	2276	WB: 1:1.000
anti-p38 MAPK	Cell Signaling	9212	WB: 1:1.000
anti-p44/42 MAPK (Erk1/2)	Cell Signaling	9102	WB: 1:1.000
anti-p70S6 Kinase	Cell Signaling	2708	WB: 1:1.000
Anti-PDGFRa	R&D Systems	AF1062	IF: 1:100
anti-Phospho-4E-BP1	Cell Signaling	2855	WB: 1:1.000
anti-Phospho-Akt	Cell Signaling	2965	WB: 1:1.000
anti-Phospho-AMPK-alpha	Cell Signaling	2535	WB: 1:1.000
anti-Phospho-eEF2	Cell Signaling	2331	WB: 1:1.000
anti-Phospho-mTOR	Cell Signaling	5536	WB: 1:1.000
anti-Phospho-p38 MAPK	Cell Signaling	9211	WB: 1:1.000
anti-Phospho-p44/42 MAPK (Erk1/2)	Cell Signaling	9101	WB: 1:1.000
anti-Phospho-p70S6 Kinase	Cell Signaling	9208	WB: 1:1.000
anti-Phospho-p70S6 Kinase	Cell Signaling	9234	WB: 1:1.000
anti-Phospho-Raptor	Cell Signaling	2083	WB: 1:1.000
anti-Phospho-Rictor	Cell Signaling	3806	WB: 1:1.000
anti-Puromycin	Sigma Aldrich	MABE343	WB: 1:5.000
anti-Raptor	Cell Signaling	2280	WB: 1:1.000
anti-Rictor	Cell Signaling	2114	WB: 1:1.000
anti-SAPK/JNK	Cell Signaling	9252	WB: 1:1.000
Anti-TIP30	Abcam	177961	WB: 1:1.000; IF: 1:100
Anti-TIP30	Abcam	71752	WB: 1:1.000
Anti-Rabbit/Mouse IgG Alexa Fluor® 488 or secondary antibody	New England Biolabs	4412	IF: 1:200
Anti-Rabbit/Mouse IgG Alexa Fluor® 555 secondary antibody	New England Biolabs	4409	IF: 1:200
Alexa Fluor® 488 Phalloidin	Thermo Fisher Scientific	A12379	IF: 1:200
**Chemicals**			

$^{13}C_6,^{15}N_2$-L-Lysine HCl	Silantes	211604102	
$^{13}C_6,^{15}N_4$-L-Arginine HCl	Silantes	201604102	
Angiotensin II	Calbiochem	05-23-0101	
Calf Thymus DNA	Thermo Fisher Scientific	15633-019	
cOmplete™ Protease Inhibitor Cocktail	Sigma-Aldrich	11697498001	
Endothelin 1	Sigma-Aldrich	E7764	
Fetal Bovine Serum (FBS) superior	Biochrom	S0615	
Guanosine 5'-Triphosphoric Acid Disodium Salt	AppliChem	A1803	
HardSet Mounting Medium with DAPI	Vector Laboratories	VEC-H-1500	
Hoechst 33258	Sigma-Aldrich	861405	
Isolectin B4	Vector Laboratories	FL-1201	
Lipofectamine 2000 reagent	Invitrogen	11668-027	
Narciclasine	Santa Cruz	Sc-361271	
Normal goat IgG	Santa Cruz	sc-2027	
Normal mouse IgG	Santa Cruz	sc-2025	
Passive Lysis Buffer	Promega	E1941	
peqGOLD TriFast	Peqlab	30-2020	
Phenylephrine	Sigma-Aldrich	P6126	
Puromycin dihydrochloride	Sigma-Aldrich	P7255	
Tissue-Tek® O.C.T™	Sakura	4583	
FITC-conjugated wheat germ agglutinin (WGA)	Sigma-Aldrich	L4895	
**Critical Commercial Kits**			
AdEasy Adenoviral Vector System	Agilent Technologies	240009	
Duolink In Situ kit	Sigma-Aldrich	DUO92101	
Flexi rabbit reticulocyte lysate	Promega	L4540	
Fluorotect GreenLys in vitro translation Labeling System	Promega	L5001	
GeneTrans II Transfection Reagent	MoBiTec GmbH	0201B	
GlutathioneSepharose 4B	GE Healthcare	17-0756-01	
Maxima H Minus First Strand cDNA Synthesis Kit	Thermo Fisher Scientific	EP0751	
Maxima SYBR Green qPCR Master Mix	Thermo Fisher Scientific	K0253	
Micro BCA Protein Assay Kit	Thermo Fisher Scientific	23235	
Ni-NTA Magnetic Agarose beads	Qiagen	36113	
Ni-NTA Spin columns	Qiagen	31014	
PCR purification kit	Thermo Fisher Scientific	K0701	
PE Annexin V Apoptosis Detection Kit I	BD Biosciences	559763	
Protein A/G PLUS Agarose beads	Santa Cruz	sc-2003	

Renilla Luciferase Assay System	Promega	E2810	
Zeba Spin desalting columns 7K MWCO	Thermo Fisher Scientific	89882	
**Experimental Models: Cell Lines**			
COS-1	Laboratory of Kai C. Wollert	N/A	
HEK293	Leibniz Institute DSMZ	Cell Line 293 ACC: 305	
**Experimental Models: Organisms/Strains**			
E.coli: BJ5183-AD-1 Electroporation Competent Cells	Agilent Technologies	200157	
E.coli: DH5α™ Competent Cells	Thermo Fisher Scientific	18265-017	
E.coli: One Shot® BL21 Star™ (DE3) Chemically Competent	Thermo Fisher Scientific	18265-017	
E.coli: XL10-Gold Ultracompetent Cells	Agilent Technologies	200315	
Mouse: B6.129P2-Htatip2^{tm1Hx}	Laboratory of H.Xiao	[(Ito et al., 2003)]	
Mouse: C57BL/6N	The Jackson Laboratory	JAX: 005304	
Mouse: C57BL/mdx	Laboratory of O. J. Müller		
**Recombinant DNA**			
Plasmid: pcDNA3.1/V5-eEF1A1-HIS	This paper	N/A	
Plasmid: pcDNA3.1/V5-eEF1A1-ΔAS1-240-HIS (ΔDom1)	This paper	N/A	
Plasmid: pcDNA3.1/V5-eEF1A1-ΔAS241-336-HIS (ΔDom2)	This paper	N/A	
Plasmid: pcDNA3.1/V5-eEF1A1-ΔAS336-462-HIS (ΔDom3)	This paper	N/A	
Plasmid: pcDNA3.1/V5-eEF1A2-HIS	This paper	N/A	
Plasmid: pcDNA3.1/V5-TIP30-HIS	This paper	N/A	
Plasmid: pcDNA3.1/V5-TIP30-Δ102-107-HIS	This paper	N/A	
Plasmid: pcDNA3.1/V5-TIP30-ΔC15-HIS	This paper	N/A	
Plasmid: pcDNA3.1/V5-TIP30-ΔN25-HIS	This paper	N/A	
Plasmid: pcDNA3.1/V5-TIP30-ΔN52-HIS	This paper	N/A	
Plasmid: pds-CMV$_{enh}$-MLC260-TIP30	This paper	N/A	
Plasmid: pds-TntT-TIP30	This paper	N/A	
Plasmid: pGEX-4T1-eEF1A1	This paper	N/A	
Plasmid: pGEX-4T1-eEF1A2	This paper	N/A	
Plasmid: pGEX-4T1-TIP30	This paper	N/A	

Plasmid: pGEX-4T1-TIP30-AS1-130	This paper	N/A	
Plasmid: pGEX-4T1-TIP30-AS1-180	This paper	N/A	
Plasmid: pGEX-4T1-TIP30-AS1-230	This paper	N/A	
Plasmid: pGEX-4T1-TIP30-AS1-50	This paper	N/A	
Plasmid: pshuttleCMV-eEF1A1-Myc	This paper	N/A	
Plasmid: pshuttleCMV-hnRNPA2/B1-Myc	This paper	N/A	
Plasmid: pshuttleCMV-Nucleolin-Myc	This paper	N/A	
Plasmid: pshuttleCMV-Rps3a-Myc	This paper	N/A	
Plasmid: pshuttleCMV-TIP30	This paper	N/A	
Plasmid: pshuttleCMV-TIP30-HIS	This paper	N/A	
Vector: pcDNA3.1/V5-His A	Thermo Fisher Scientific	V81020	
Vector: pDP9rs	Laboratory of O. J. Müller	N/A	
Vector: pds-CMV$_{enh}$-MLC260 (AAV9)	Laboratory of O. J. Müller	N/A	
Vector: pds-TntT-Cre	Laboratory of O. J. Müller	N/A	
Vector: pGEX-4T1	GE Healthcare	28-9545-49	
Vector: pShuttleCMV	Agilent Technologies	240007	
Adenovirus expressing eEF1A1-Myc	This paper	N/A	
Adenovirus expressing TIP30	This paper	N/A	
Adenovirus expressing TIP30-HIS	This paper	N/A	
Adenovirus expressing β-Gal (Ad.Control)	Previous work	[(Froese et al., 2011)]	
cDNA clone eEF1A1	GE Healthcare	MRN1768-202843669	
cDNA clone eEF1A2	GE Healthcare	MRN1768-202780416	
cDNA clone eEF1B2	GE Healthcare	ERN1146-202843321	
cDNA clone hnRNPA2/B1	GE Healthcare	MHS6278-202826002	
cDNA clone Nucleolin	GE Healthcare	MRN1768-202780611	
cDNA clone Rps3a	GE Healthcare	MRN1768-202843526	
**Sequence-Based Reagents**			
Silencer® Negative Control No. 1 siRNA	Thermo Fisher Scientific	AM4611	
siRNA eEF1A1	Sigma Aldrich	SASI_Rn02_00269532	

Primer	Forward (5'-to-3' sequence)	Reverse (5'-to-3' sequence)	
Cloning primer for human hnRNPA2/B1-Myc	TATAGGTACCATGGAGAGAGAAAAGGAACA	TTAAGCGGCCGCCTACAGATCTTCTTCAGAAATAAGTTTTTGTTCTTGGACCGTAGTTAGAAGGT	
Cloning primer for rat eEF1A1-His	GCGATCTAGAATGGGAAAGGAAAAGACTCA	GCGAACCGGTTTTAGCCTTCTGAGCTTTCT	
Cloning primer for rat eEF1A1-Myc	TGACGGTACCATGGGAAAGGAAAAGACTCA	ATATCTCGAGCTACAGATCTTCTTCAGAAATAAGTTTTTGTTCTTTAGCCTTCTGAGCTTTCT	
Cloning primer for rat eEF1A1-Δ 241-336-His (ΔDom2) I	TGACGGTACCATGGGAAAGGAAAAGACTCA	ATATCTCGAGACGAGTTGGCGGCAGAAT	
Cloning primer for rat eEF1A1-Δ 241-336-His (ΔDom2) II	ATATCTCGAGGCAGCTGGCTTCACTGCT	ATATACCGGTTTTAGCCTTCTGAGCTTTCTG	
Cloning primer for rat eEF1A1-Δ 336-462-His (ΔDom3)	ATATCTCGAGACGAGTTGGCGGCAGAAT	ATATCTCGAGTTCCATTGGTGGGTCATTT	
Cloning primer for rat eEF1A1-Δ1-240-His (ΔDom1)	TTATGGTACCATGCCAACTGACAAGCCTCTG	ATATACCGGTTTTAGCCTTCTGAGCTTTCTG	
Cloning primer for rat eEF1B2-His	ATATGAATTCATGGGTTTCGGAGACCTGA	ATATCTCGAGGATTTTGTTAAAAGCAGCCACAT	
Cloning primer for rat GST-eEF1A1	TCTCGAATTCGGAAAGGAAAAGACTCA	ATATGTCGACAGGTGTTAGGGATAATAT	
Cloning primer for rat GST-eEF1B2	ATATCTGAATTCGGTTTCGGAGACCTGAAAA	ATATCTCTCGAGTTAGATTTTGTTAAAAGCAGCCA	

Cloning primer for rat Nucleolin-Myc	GCGCAGATCT ATGGTGAAAC TCGCAAAGGC	GCGCCTCGA GCTACAGAT CTTCTTCAGA AATAAGTTTT TGTTCTTCAA ACTTCGTCTT CTTTC	
Cloning primer for rat Rps3a-Myc	TATAGGTACC ATGGCGGTCG GGAAGAACAA	ATATCTCGA GCTACAGAT CTTCTTCAGA AATAAGTTTT TGTTCAACG GATTCTTGG ACTGGTG	
Cloning primer for rat TIP30 (AAV9-TIP30)	ATATGGATCC ACCGGTCGCC ACCATGGCGG ACAAGGAAAC ACTGCT	GCGCTGTAC ATCAGTTATC TAGATCCGG TGGA	
Cloning primer for rat TIP30 (Ad.TIP30)	AGAGGGTACC ATGGCGGACA AGGAAACAC	TTGGCTCGA GAGTTTGGG TCCGTCGCT GT	
Cloning primer for rat TIP30 (GST-TIP30)	ATATGGATCC GCGGACAAGG AAACACTGCT	TTGGGAATT CCGGCATGG TCACAGTTT G	
Cloning primer for rat TIP30 (GST-TIP30-AS1-130)	ATATGGATCC GCGGACAAGG AAACACTGCT	GTGTGAATT CCAGCAAGT TGAAATGTTT GC	
Cloning primer for rat TIP30 (GST-TIP30-AS1-180)	ATATGGATCC GCGGACAAGG AAACACTGCT	GTGTGAATT CGCCTGGAC GAGACTGTT GCC	
Cloning primer for rat TIP30 (GST-TIP30-AS1-230)	ATATGGATCC GCGGACAAGG AAACACTGCT	GTGTGAATT CGATGGCCT TATTTTCCAG AA	
Cloning primer for rat TIP30 (GST-TIP30-AS1-50)	ATATGGATCC GCGGACAAGG AAACACTGCT	GTGTGAATT CAATGAGCG TTACTTTGGA AA	
Cloning primer for rat TIP30-His	AGAGGGTACC CAATGGCGGA CAAGGAAACA	ATCGACCGG TCAGTTTGG GTCCGTCGC TGT	
Cloning primer for rat TIP30-Δ102-107-His I	AGAGGGTACC CAATGGCGGA CAAGGAAACA	ATATCTCGA GCGCTCCAG CTTTTCTCCT	

Cloning primer for rat TIP30-Δ102-107-His II	ATATCTCGAGCGAGATTATGTGCTGAAGT	ATATACCGGTCAGTTTGGGTCCGTCG	
Cloning primer for rat TIP30-ΔC15-His	AGAGGGTACCCAATGGCGGACAAGGAAACA	ATATACCGGTCTTATTTTCCAGAAGTTCCATTT	
Cloning primer for rat TIP30-ΔN25-His	TTATGGTACCATGGCCAGCGGTGAAACCGG	ATATACCGGTCAGTTTGGGTCCGTCG	
Cloning primer for rat TIP30-ΔN52-His	TTATGGTACCATGAGGAAGCTCACATTTGA	ATATACCGGTCAGTTTGGGTCCGTCG	
qRT-PCR primer for human GAPDH	TCGACAGTCAGCCGCATCTTCTTT	ACCAAATCCGTTGACTCCGACCTT	
qRT-PCR primer for human TIP30	ACCGGCAGAGTGCTCTTAAA	TCTCGGTCAACACGAACAAA	
qRT-PCR primer for mouse Acta1	TGTCGTCCCAGTTGGTGATA	ACCGCTCTTGTGTGTGACAA	
qRT-PCR primer for mouse eEF1A1	CGGCCACCTGATCTACAAAT	CACGCTCAGCTTTCAGTTTG	
qRT-PCR primer for mouse eEF1A2	TGAGATGGTCCCTGGAAAAC	GCTTTCTGAGCCTTCTGTGC	
qRT-PCR primer for mouse GAPDH	ACCCAGAAGACTGTGGATGG	CACATTGGGGGTAGGAACAC	
qRT-PCR primer for mouse L7	TGGAACCATGGAGGCTGT	TCTCAGTGCGGTACATCTGC	
qRT-PCR primer for mouse Myh6	ACTGTGGTGCCTCGTTCC	GCCTCTAGGCGTTCCTTCTC	
qRT-PCR primer for mouse Myh7	AGGCAAGGCAAAGAAAGGCTCATC	GCGTGGAGCGCAAGTTTGTCATAA	
qRT-PCR primer for mouse TIP30	GAAGTGGAAGCCAAGGTTGA	CTGGGACTCACCAGGTTGTT	
qRT-PCR primer for rat GAPDH	ACCACCATGGAGAAGGCTGG	CTCAGTGTAGCCCAGGATGC	
qRT-PCR primer for rat L7	GAAGCTCATCTATGAGAAGGC	AAGACGAAGGAGCTGCAGAAC	

qRT-PCR primer for rat TIP30	CGACCGAGATTATGTGCTGA	GGACGAGACTCTTGCCTGTC	
qRT-PCR primer for renilla	CCAGGGATTTCAGTCGATGT	AATCTCACGCAGGCAGTTCT	
qRT-PCR primer for mouse Rheb1	CCATGGCAAGTTGTTGGATATG	TCTTCATAGCTGATCACCCTTTC	
qRT-PCR primer for mouse Hrd1	CCAACATCTCCTGGCTCTTCCA	CAGGATGCTGTGATAAGCGTGG	
qRT-PCR primer for mouse Xbp1	CTCACGGCCTTGTGGTTGA	TCCATTCCCAAGCGTGTTC	
qRT-PCR primer for mouse Manf	TCAATGAGGTGTCGAAGCCC	GTCCACTGTGCTCAGGTCAA	
**Software and Algorithms**			
Graph Pad Prism 6	Graph Pad software	N/A	
ImageJ (Schneider et al., 2012)	National Institutes of Health, USA	N/A	
Quantity One	Bio-Rad	N/A	
Ion Wizard software	IonOptix		

Appendix Table S5: Statistics

Figure		Exact *P*-value	Statistical test
**1**	B	WT TAC/KO TAC: *=0.0475; WT TAC/Het TAC: ****< 0.0001	one-way ANOVA followed by the Sidak´s multiple comparisons test
	C	WT TAC/Het TAC: ****< 0.0001	one-way ANOVA followed by the Sidak´s multiple comparisons test
	D	WT TAC/Het TAC: *=0.0273	one-way ANOVA followed by the Sidak´s multiple comparisons test
	E	WT Sham/WT TAC: **=0.0069; WT TAC/KO TAC: ***=0.0003; WT TAC/Het TAC: ****< 0.0001	one-way ANOVA followed by the Sidak´s multiple comparisons test
	F	WT TAC/ Het TAC: *= 0.0161	one-way ANOVA followed by the Sidak´s multiple comparisons test
	G	WT Sham/WT TAC: *=0.0279; WT TAC/Het TAC: **=0.0051	one-way ANOVA followed by the Sidak´s multiple comparisons test
	I	WT TAC/Het TAC: **=0.0081	one-way ANOVA followed by the Sidak´s multiple comparisons test
	L	WT Sham/WT TAC: *=0.0479; WT TAC/Het TAC: *=0.0292	one-way ANOVA followed by the Sidak´s multiple comparisons test
	N	WT TAC/Het TAC: **=0.0055	one-way ANOVA followed by the Sidak´s multiple comparisons test
	Q	WT AAV-Con TAC/Het AAV-Con TAC: *=0.0133; Het AAV-Con TAC/Het AAV-TIP30 TAC: *=0.0257; WT AAV-Con Sham/WT AAV-Con TAC: ***=0.0008	one-way ANOVA followed by the Sidak´s multiple comparisons test
	R	WT AAV-Con TAC/Het AAV-Con TAC: *=0.0163; Het AAV-Con TAC/Het AAV-TIP30 TAC: *=0.0354	one-way ANOVA followed by the Sidak´s multiple comparisons test
**2**	B	Ad.Con ET-1/Ad.TIP30 ET-1: *=0.0348; Ad.Con/Ad.TIP30: ***=0.0001; Ad.Con PE/Ad.TIP30 PE: ***=0.0002; Ad.Con/Ad.Con FBS: ***=0.0008; Ad.Con/Ad.Con PE: ****<0.0001	one-way ANOVA followed by the Sidak´s multiple comparisons test
	C	Ad.Con/Ad.Con PE: *=0.0162; Ad.Con PE/Ad.TIP30 PE: ****<0.0001	one-way ANOVA followed by the Sidak´s multiple comparisons test
	D	Ad.Con/Ad.Con ET-1: *=0.0152; Ad.Con ET-1/Ad.TIP30 ET-1: *=0.0435; Ad.Con PE/Ad.TIP30 PE: **=0.0014; Ad.Con/Ad.Con PE: ****<0.0001	one-way ANOVA followed by the Sidak´s multiple comparisons test
	H	AAC-Con Sham/AAV-Con TAC: *=0.0196	one-way ANOVA followed by the Sidak´s multiple comparisons test
	I	AAC-Con Sham/AAV-Con TAC: *=0.0118; AAV-Con TAC/AAV-TIP30 TAC: *=0.0438	one-way ANOVA followed by the Sidak´s multiple comparisons test

	J	AAV-Con 2wk TAC/AAV-TIP30 2wk TAC: **=0.0028; AAV-Con 4wk TAC/AAV-TIP30 4wk TAC: **=0.0048	one-way ANOVA followed by the Sidak´s multiple comparisons test
**3**	J	eEF1A1 mRNA expression neonatal/adult sham: *=0.0468; eEF1A1 mRNA expression adult sham/adult TAC: *=0.0180; eEF1A2 mRNA expression neonatal/adult sham: ***=0.0003	one-way ANOVA followed by the Sidak´s multiple comparisons test
**4**	C	Ad.Con PE/Ad.TIP30 PE: *=0.0131	two-sided Student's t -test
	E	Ad.Con/Ad.Con PE: ****<0.0001; Ad.Con PE/Ad.TIP30 PE: ****<0.0001	one-way ANOVA followed by the Sidak´s multiple comparisons test
	G	WT Sham/WT TAC: *=0.0355; WT TAC/Het TAC: *=0.0277	one-way ANOVA followed by the Sidak´s multiple comparisons test
**5**	D	ratio TIP30/GAPDH expression: ****<0.0001; ratio eEF1A1/GAPDH expression: **= P = 0.0083	two-sided Student's t -test
	H	ratio TIP30/Actin expression: *=0.0228; ratio TIP30/eEF1A1 expression: **=0.0039	two-sided Student's t -test
	J	ratio TIP30/Actin expression: ***=0.0005; ratio TIP30/eEF1A1 expression: **=0.0058	two-sided Student's t -test
**6**	A	Tip30 mRNA expression: ***=0.0003; ratio TIP30/eEF1A1 mRNA expression: *=0.0137	two-sided Student's t -test
	B	Tip30 mRNA expression: **=0.0077; ratio TIP30/eEF1A1 mRNA expression: *= *P* = 0.0492	two-sided Student's t -test
	C	Tip30 mRNA expression: ****<0.0001; eEF1A1 mRNA expression: **= *P* = 0.0020; ratio TIP30/eEF1A1 mRNA expression: ****<0.0001	two-sided Student's t -test
	E	AAV-Con 3mo/AAV-TIP30 3mo: *=0.0132; AAV-Con 9mo/AAV-TIP30 9mo: **=0.0017	one-way ANOVA followed by the Sidak´s multiple comparisons test
	F	AAV-Con/AAV-TIP30: *=0.0116	two-sided Student's t -test
**7**	B	Ad.Con/Ad.Con PE: **=0.0028; Ad.Con PE/Ad.TIP30 PE: *=0.0491	one-way ANOVA followed by the Sidak´s multiple comparisons test
	C	WT ET-1/Het ET-1: *=0.0143; Het ET-1/Het ET-1 + Narci: ***=0.0001	one-way ANOVA followed by the Sidak´s multiple comparisons test
	E	WT TAC/Het TAC: ***=0.0002	one-way ANOVA followed by the Sidak´s multiple comparisons test
	F	WT TAC/Het TAC: ***=0.0007; Het TAC/Het TAC Narci: *P*=0.09	one-way ANOVA followed by the Sidak´s multiple comparisons test
	G	WT TAC/Het TAC: **=0.0052; Het TAC/Het TAC Narci: *=0.0235	one-way ANOVA followed by the Sidak´s multiple comparisons test
	H	Het TAC/Het TAC Narci: **=0.0063	one-way ANOVA followed by the Sidak´s multiple comparisons test
	I	Het TAC/Het TAC Narci: *=0.0102; WT TAC/Het TAC: **=0.0086	one-way ANOVA followed by the Sidak´s multiple comparisons test
**EV1**	A	WT TAC/ Het TAC: **=0.0100	one-way ANOVA followed by the Sidak´s multiple comparisons test

	B	WT TAC/ Het TAC: *=0.0344	one-way ANOVA followed by the Sidak´s multiple comparisons test
	C	WT TAC/ Het TAC: ***=0.0003	one-way ANOVA followed by the Sidak´s multiple comparisons test
	E	WT Sham/WT TAC: ***=0.0002; Het Sham/Het TAC: ****<0.0001	one-way ANOVA followed by the Sidak´s multiple comparisons test
	F	WT TAC/Het TAC: *=0.0214; WT Sham/WT TAC: ***=0.0003	one-way ANOVA followed by the Sidak´s multiple comparisons test
**EV2**	A	WT /Het: ***=0.0004	two-sided Student's t -test
	C	WT /Het: ***=0.0002	two-sided Student's t -test
	G	WT /Het: *=0.0478	two-sided Student's t -test
**EV3**	B	P-p70S6 (S371) expression: WT sham/Het sham: *=0.0485; WT TAC/Het TAC: *=0.0169<br>P-p70S6 (T389) expression: WT TAC/Het TAC: *=0.0298; WT Sham/WT TAC: ****<0.0001<br>P-mTOR expression: WT TAC/Het TAC: *=0.0120<br>P-AMPKα expression: WT TAC/Het TAC: **=0.0029<br>P-SAPK/JNK expression: WT Sham/WT TAC: **=0.0087<br>P-ERK1/2 expression: WT TAC/Het TAC: *=0.0351; WT sham/Het sham: **=0.0035	one-way ANOVA followed by the Sidak´s multiple comparisons test
**EV4**	D	Ad.Con PE/Ad.TIP30 PE: *=0.0280; Ad.Con/Ad.Con PE: **=0.0038	one-way ANOVA followed by the Sidak´s multiple comparisons test
	E	Ad.Con PE/Ad.TIP30 PE: *=0.0190; Ad.Con/Ad.Con PE: *=0.0017	one-way ANOVA followed by the Sidak´s multiple comparisons test
	G	AAV-Con Sham/AAV-Con TAC: *=0.0138; AAV-Con TAC/AAV-TIP30 TAC: **0.0011	one-way ANOVA followed by the Sidak´s multiple comparisons test
**EV5**	A	WT/Het: *=0.0393	one-way ANOVA followed by the Sidak´s multiple comparisons test
	B	Hrd1 expression: WT TAC/KO TAC: **=0.0016<br>Xbp1 expression: WT TAC/KO TAC: *=0.0475<br>Manf expression: WT TAC/KO TAC: **=0.0019<br>Rheb1 expression: WT TAC/Het TAC: **=0.0017; WT TAC/KO TAC: ***=0.0009	one-way ANOVA followed by the Sidak´s multiple comparisons test
	D	Ad.Con Scrl/Ad.TIP30 Scrl: **= 0.0067; Ad.Con Scrl/Ad.Con eEF1A1: **= 0.0080	one-way ANOVA followed by the Sidak´s multiple comparisons test
	E	Ad.Con PE/Ad.Con PE +Narci: *=0.0348; Ad.Con/Ad.Con PE: **=0.0061; Ad.Con PE/Ad.TIP30 PE: ****<0.0001; Ad.Con PE +Narci/Ad.TIP30 PE +Narci: *P*=0.08	one-way ANOVA followed by the Sidak´s multiple comparisons test
	F	TIP30-HIS ΔN52/TIP30-HIS full: *=0.0394; BSA/TIP30-HIS full: ***=0.0002	one-way ANOVA followed by the Sidak´s multiple comparisons test

**Appendix S3**	C	AAV-TIP30/AAV-TIP30 +Dobutamine: *=0.0382; AAV-Con/AAV-Con +Dobutamine: *P*=0.8132	one-way ANOVA followed by the Sidak´s multiple comparisons test

Appendix Table S6: *N* values

Figure		*N*
**1**	B	WT sham=6; Het Sham=8; KO Sham=6; WT TAC=18; Het TAC=17; KO TAC=11
	C	WT sham=6; Het Sham=8; KO Sham=6; WT TAC=18; Het TAC=17; KO TAC=11
	D	WT sham=6; Het Sham=8; KO Sham=6; WT TAC=16; Het TAC=15; KO TAC=14
	E	WT sham=7; Het Sham=8; KO Sham=6; WT TAC=16; Het TAC=15; KO TAC=14
	F	WT sham=4; Het Sham=5; WT TAC=11; Het TAC=7
	G	WT sham=4; Het Sham=5; WT TAC=11; Het TAC=8
	H	WT sham=5; Het Sham=6; WT TAC=10; Het TAC=10
	I	WT sham=5; Het Sham=6; WT TAC=10; Het TAC=10
	K	WT sham=3; Het Sham=3; WT TAC=5; Het TAC=5
	L	WT sham=4; Het Sham=4; WT TAC=7; Het TAC=11
	N	WT sham=5; Het Sham=6; WT TAC=7; Het TAC=6
	Q	WT AAV-Con Sham=5; Het AAV-Con Sham=3; WT AAV-TIP30 Sham=5; Het AAV-TIP30 Sham=6; WT AAV-CON TAC=5, Het AAV-Con TAC=6; WT AAV-TIP30 TAC=9; Het AAV-TIP30 TAC=10
	R	WT AAV-Con Sham=5; Het AAV-Con Sham=3; WT AAV-TIP30 Sham=5; Het AAV-TIP30 Sham=6; WT AAV-CON TAC=5, Het AAV-Con TAC=6; WT AAV-TIP30 TAC=9; Het AAV-TIP30 TAC=10
**2**	B	Ad. Con=8; Ad.TIP30=8; Ad.Con ET-1=8; Ad.TIP30 ET-1=8; Ad.Con FBS=6; Ad.TIP30 FBS=6, Ad.Con PE=8; Ad.TIP30 PE=8
	C	Each group *N*=9
	D	Each group *N*=3
	E	Each group *N*=7
	H	AAV-Con Sham=8; AAV-TIP30 Sham=8; AAV-Con TAC=13; AAV-TIP30 TAC=11
	I	AAV-Con Sham=4; AAV-TIP30 Sham=3; AAV-Con TAC=5; AAV-TIP30 TAC=5
	J	AAV-Con Sham=10 each time point; AAV-TIP30 Sham=10 each time point; AAV-Con TAC 2wk=14; AAV-Con TAC 4wk=13; AAV-Con TAC 6wk=13; AAV-TIP30 TAC 2wk=14; AAV-TIP30 TAC 4wk=14; AAV-TIP30 TAC 6wk=13
**3**	J	Neonatal=3; Adult Sham=3; Adult TAC=4
**4**	C	Each group *N*=3
	E	Ad.Con=3; Ad.TIP30=3; Ad.Con PE=4; Ad.TIP30=4
	G	WT Sham=7; Het Sham=8; WT TAC=11; Het TAC=12
**5**	A	Each group *N*=4
	D	Sham=4; TAC 2wk= 8
	F	Sham=4; TAC 6wk= 8
	H	WT TAC=3; Het TAC 2wk= 4
	J	Each group *N*=4
**6**	A	Control=6; Failing=8

	B	Control=4; HCM=8
	C	WT=3; MDX=4
	E	AAV-Con baseline=7; AAV-TIP30 baseline=7; AAV-Con 1mo=7; AAV-TIP30 1mo=6; AAV-Con 3mo=7; AAV-TIP30 3mo=7; AAV-Con 6mo=7; AAV-TIP30 6mo=7; AAV-Con 9mo=6; AAV-TIP30 9mo=6
	F	AAV-Con=6; AAV-TIP30=5
**7**	B	Each group *N*=3
	C	Each group *N*=6
	E	WT Sham=6; WT TAC=8; Het TAC=9; WT TAC +Narci=10; Het TAC +Narci=6
	F	WT Sham=6; WT TAC=8; Het TAC=9; WT TAC +Narci=10; Het TAC +Narci=6
	G	Each group *N*=5
	H	WT Sham=6; WT TAC=8; Het TAC=9; WT TAC +Narci=10; Het TAC +Narci=6
	I	WT Sham=2; Het Sham=2; WT TAC=3; Het TAC=3; WT TAC +Narci=4; Het TAC +Narci=4
**EV1**	A	WT sham=7; Het Sham=8; KO Sham=6; WT TAC=16; Het TAC=17; KO TAC=14
	B	WT sham=7; Het Sham=8; KO Sham=6; WT TAC=16; Het TAC=17; KO TAC=14
	C	WT sham=7; Het Sham=8; KO Sham=6; WT TAC=16; Het TAC=17; KO TAC=14
	D	WT sham=7; Het Sham=8; KO Sham=6; WT TAC=16; Het TAC=17; KO TAC=14
	E	WT Sham=5; Het Sham=5; WT TAC=5; Het TAC=7
	F	WT Sham=4; Het Sham=5; WT TAC=11; Het TAC=8
	G	Each group *N*=5 mice
	I	WT sham=4; Het Sham=4; KO Sham=2; WT TAC=5; Het TAC=5; KO TAC=4
	J	WT sham=3; Het Sham=3; WT TAC=5; Het TAC=5
**EV2**	A	Each group *N*=4
	B	Each group *N*=4
	C	Each group *N*=4
	D	Each group *N*=4
	E	Each group *N*=4
	G	Each group *N*=4
**EV3**	B	p38, SAPK/JNK, ERK1/2, AKT, p70S6 kinase, 4E-BP1, eEF2: WT sham=3; Het Sham=3; WT TAC=4; Het TAC=4<br>mTOR, AMPKα: WT Sham=2, Het Sham=2, WT TAC=3, Het TAC=3
**EV4**	D	Ad.Con=7; Ad.TIP30=7; Ad.Con PE=8; Ad.TIP30=8
	E	Ad.Con=7; Ad.TIP30=7; Ad.Con PE=8; Ad.TIP30=8
	G	AAV-Con Sham=3; AAV-TIP30 Sham=3; AAV-Con TAC=7; AAV-TIP30 TAC=6
**EV5**	A	WT=3; Het=4; KO=3
	B	WT sham=6; Het Sham=6; KO Sham=4; WT TAC=10; Het TAC=10; KO TAC=5
	D	Ad.Con si-Scrl=12; Ad.TIP30 si-Scrl=12; Ad.Con si-eEF1a1=9; Ad.TIP30 si-eEF1A1=9

	E	Each group *N*=3
	F	Each group *N*=4
**Appendix S2**		Each group *N*=2
**Appendix S2**	B	AAV-Con=6; AAV-TIP30=5; AAV-Con +Dobutamine=6; AAV-TIP30 +Dobutamine=5
	C	AAV-Con=6; AAV-TIP30=5; AAV-Con +Dobutamine=6; AAV-TIP30 +Dobutamine=5
