# Supplementary material for: TIP30 counteracts cardiac hypertrophy and failure by inhibiting translational elongation
Source: EMBO Mol Med. 2019 Aug 30;11(10):e10018. doi: 10.15252/emmm.201810018 (PMC6783653; doi:10.15252/emmm.201810018)
Supplement: Supplementary file 2 — Expanded View Figures PDF [file EMMM-11-e10018-s002.pdf]

Expanded View Figures

Figure EV1. Additional characterization of TIP30-deficient mice during transverse aortic constriction (TAC).

A–D Fractional area change (A), left ventricular end-diastolic area (LVEDA; B), average diastolic wall thickness (C), and heart rate (D) as determined by echocardiography 2 weeks after sham or TAC surgery in *Tip30* Het, KO, or WT mice (N = 6–17 mice/group). *$P <$ 0.05, **$P <$ 0.01, ***$P <$ 0.001. One-way ANOVA with Sidak's multiple comparisons test.

E Doppler measurements of right (RCA) to left (LCA) carotid artery blood flow in TIP30 Het, or WT mice 2 days after TAC surgery (N = 5–7 mice/group). ***$P <$ 0.001, ****$P <$ 0.0001. One-way ANOVA with Sidak's multiple comparisons test.

F Quantification of maximal systolic pressure in indicated mice 6 weeks after TAC or sham surgery (N = 4–18 mice/group). *$P <$ 0.05, ***$P <$ 0.001. One-way ANOVA with Sidak's multiple comparisons test.

G Ventricular cardiomyocyte sarcomere shortening at 1, 2, and 4 Hz of isolated adult cardiomyocytes from TIP30 Het, or WT mice 2 weeks after TAC surgery (N = 5 mice/group).

H, I Microscopy images of heart sections of indicated mice 6 weeks after TAC surgery stained for PDGFRα (green), WGA (red), and DAPI (blue) (scale bar: 50 μm) and quantification of PDGFRα-positive cells per myocyte (N = 2–5 mice/group, I).

J Quantification of cleaved caspase 3-positive cardiomyocytes in hearts of *Tip30* Het and WT mice 6 weeks after TAC (N = 3–5 mice/group).

K Electron microscopy images of heart sections of indicated mice 6 weeks after TAC surgery (scale bar = 5 μm).

L Representative confocal microscopy images of heart sections of TIP30 Het mice subjected to 6 weeks of TAC surgery and AAV-TropT-TIP30 or AAV-control treatment. Red: TIP30, green: WGA, and blue: DAPI (scale bar: 20 μm).

M Western blot analysis for TIP30 and GAPDH from isolated adult cardiomyocytes 2 weeks after injection of AAV-control or AAV-TropT-TIP30.

Data information: Data are shown as mean ± SEM.
Source data are available online for this figure.

© 2019 The Authors

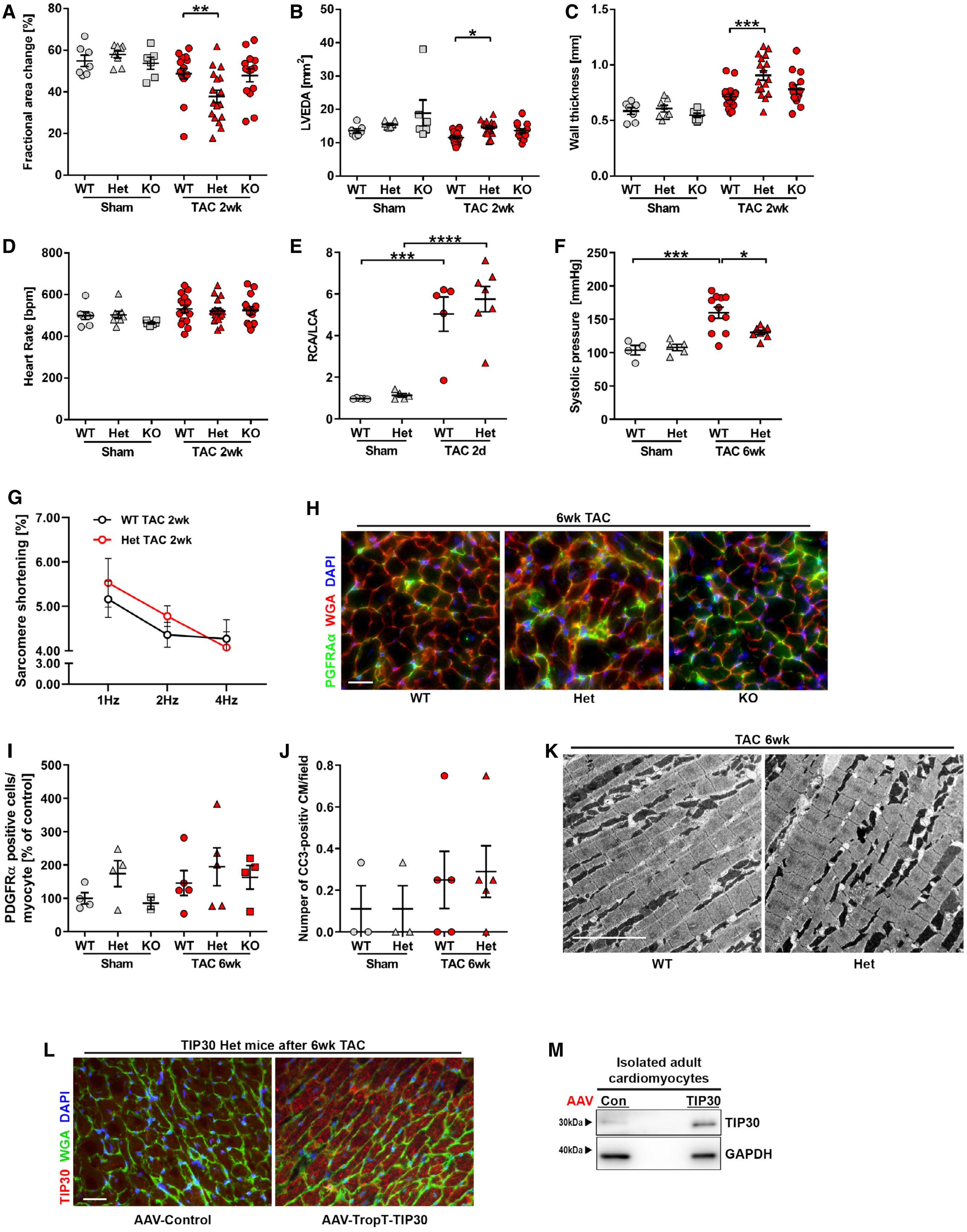

Figure EV1.

 © 2019 The Authors

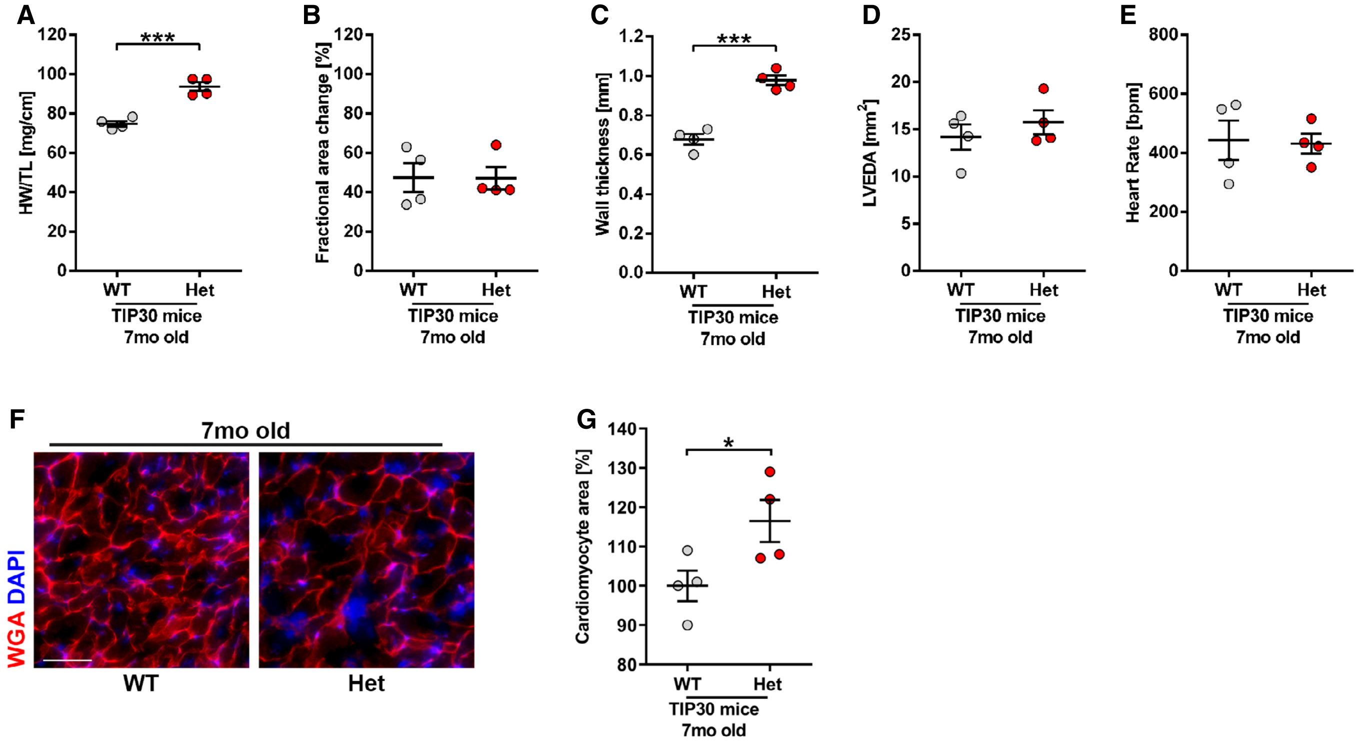

Figure EV2. Effects of TIP30 deficiency on cardiac homeostasis.

A–E Quantification of HW/Tibia length ratio (HW/TL; A), echocardiographic fractional area change (B), average diastolic wall thickness (C), left ventricular end-diastolic area (LVEDA; D), and heart rate (E) in *Tip30* Het or WT mice without additional stress stimulation at the age of 7 months (N = 4 mice/group). ***P < 0.001. Two-sided Student's t-test.

F, G Representative microscopy images of heart sections of 7-month-old TIP30 WT and Het mice stained for WGA (red) and DAPI (blue) (scale bar: 50 µm) and quantification of cell size (G). N = 4 mice/group. *P < 0.05. Two-sided Student's t-test.

Data information: Data are shown as mean ± SEM.
Source data are available online for this figure.

© 2019 The Authors

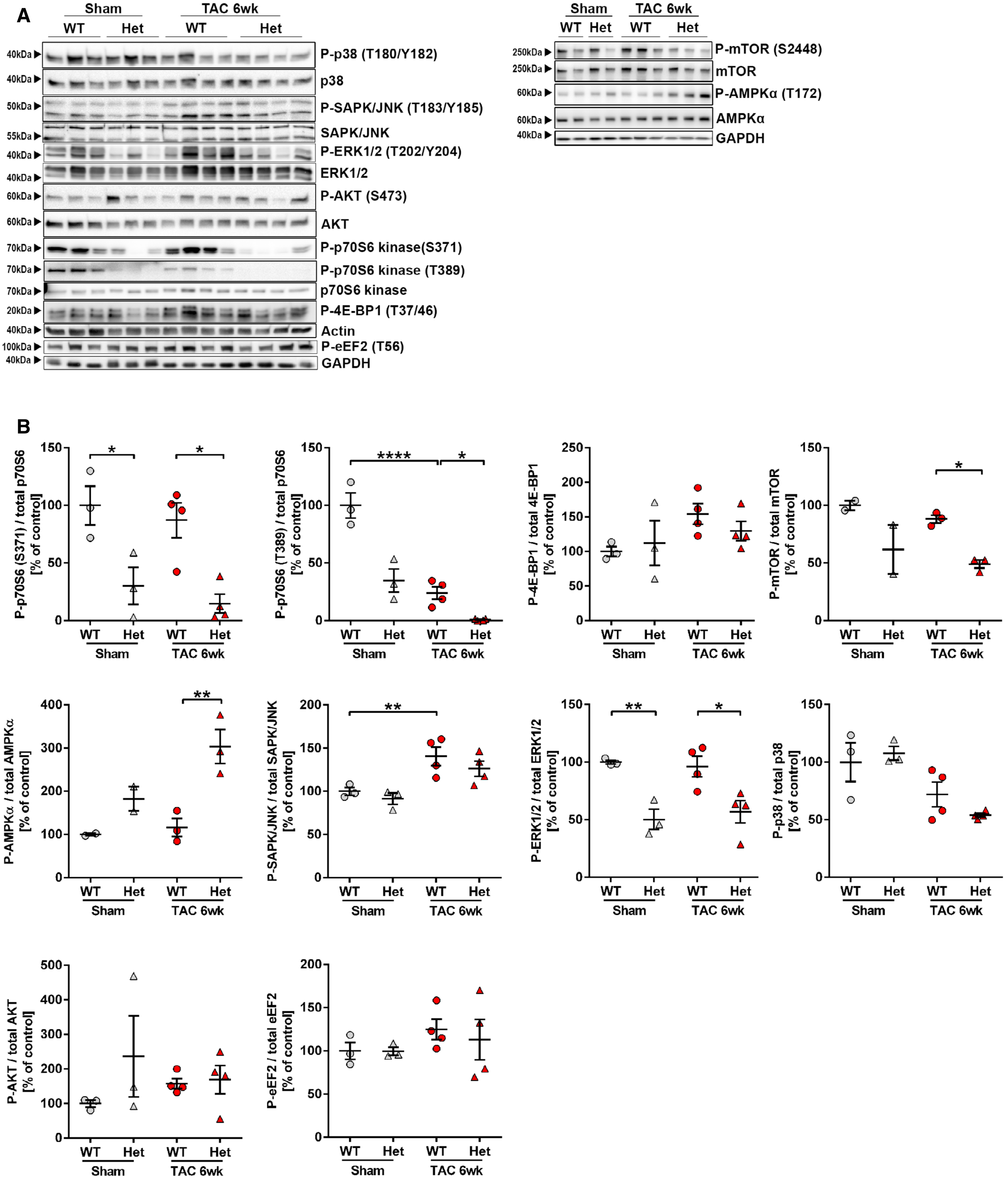

Figure EV3.

 © 2019 The Authors

Figure EV3. Interrogation of growth signaling in hearts of TIP30-deficient mice during cardiac hypertrophy.

A, B Western blot analysis of indicated proteins in hearts from TIP30 WT and Het mice 6 weeks after TAC or sham surgery (A) and their quantification (B). N = 3-4 mice/group.

Data information: Data are shown as mean ± SEM. *$P < 0.05$, **$P < 0.01$, ****$P < 0.0001$. One-way ANOVA with Sidak's multiple comparisons test.
Source data are available online for this figure.

Figure EV4. TIP30 inhibits cardiomyocyte protein synthesis at the level of translational elongation.

A Schematic representation of polysome profiling.
B Representative polysome profiles from NRCM after adenoviral transduction. Western blot analysis of rpS6 is shown to determine 40S, 60S, and 80S monosome fractions.
C–E Schematic representation of the dual-luciferase reporter assay (C). Quantification of renilla (D) and firefly (E) luciferase activity in neonatal rat cardiomyocytes (NRCM) after adenoviral transduction either with control virus (Ad. Con) or Ad.TIP30, stimulation with phenylephrine (PE) as indicated and transfection with the bi-cistronic reporter construct, as shown in (C) (N = 7–8 samples/group). *$P < 0.05$, **$P < 0.01$. One-way ANOVA with Sidak's multiple comparisons test.
F, G Western blot analysis of puromycin incorporation in hearts 6 weeks after TAC or sham surgery in AAV-Con or AAV-TIP30-treated C57BL/6 WT mice and their quantification (N = 3–7 mice/group). All lanes were run on the same gel, but were noncontiguous as indicated by separate boxes. Puromycin was injected 3 h prior to sacrifice. *$P < 0.05$, **$P < 0.01$. One-way ANOVA with Sidak's multiple comparisons test.

Data information: Data are shown as mean ± SEM.
Source data are available online for this figure.

© 2019 The Authors

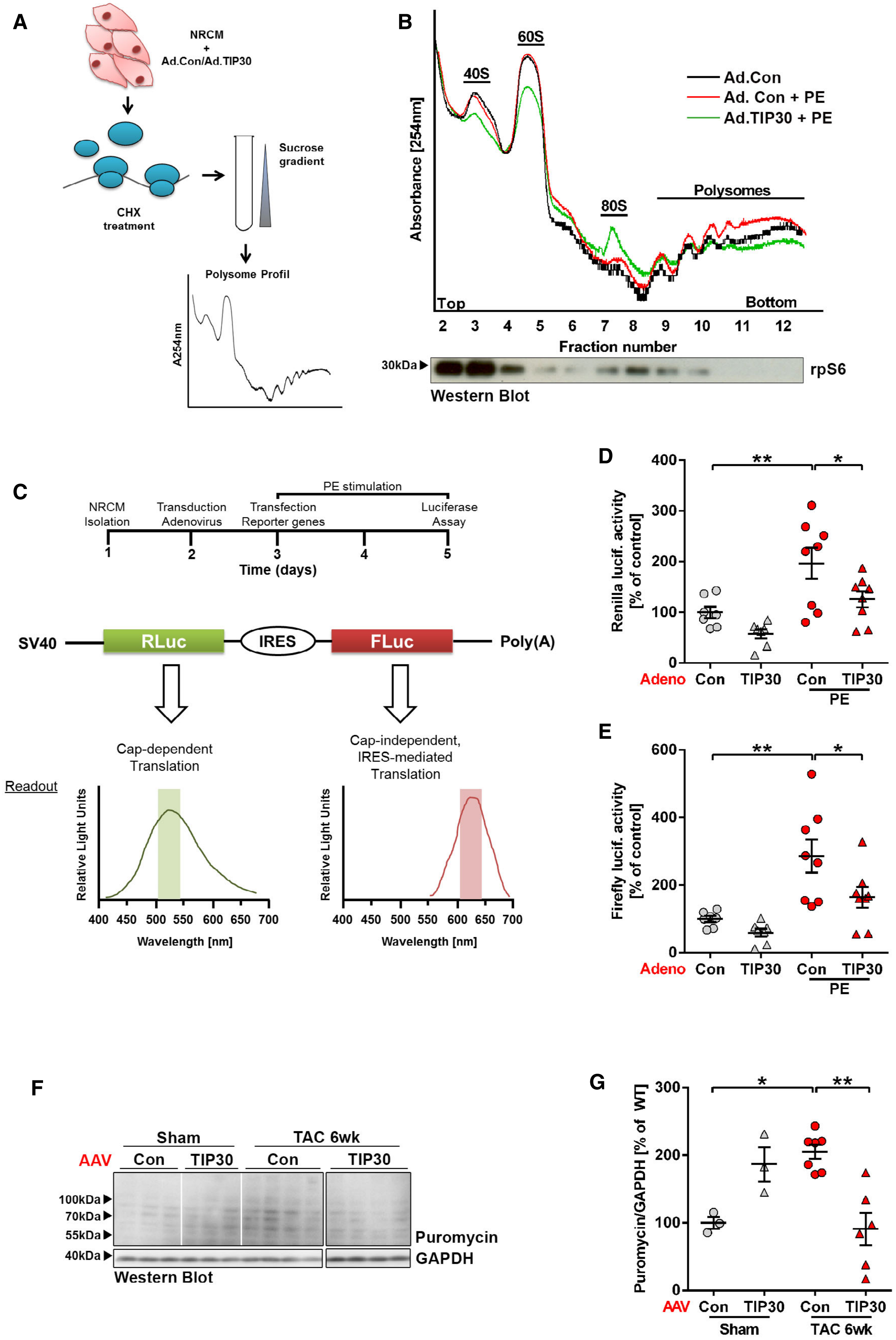

Figure EV4.

© 2019 The Authors

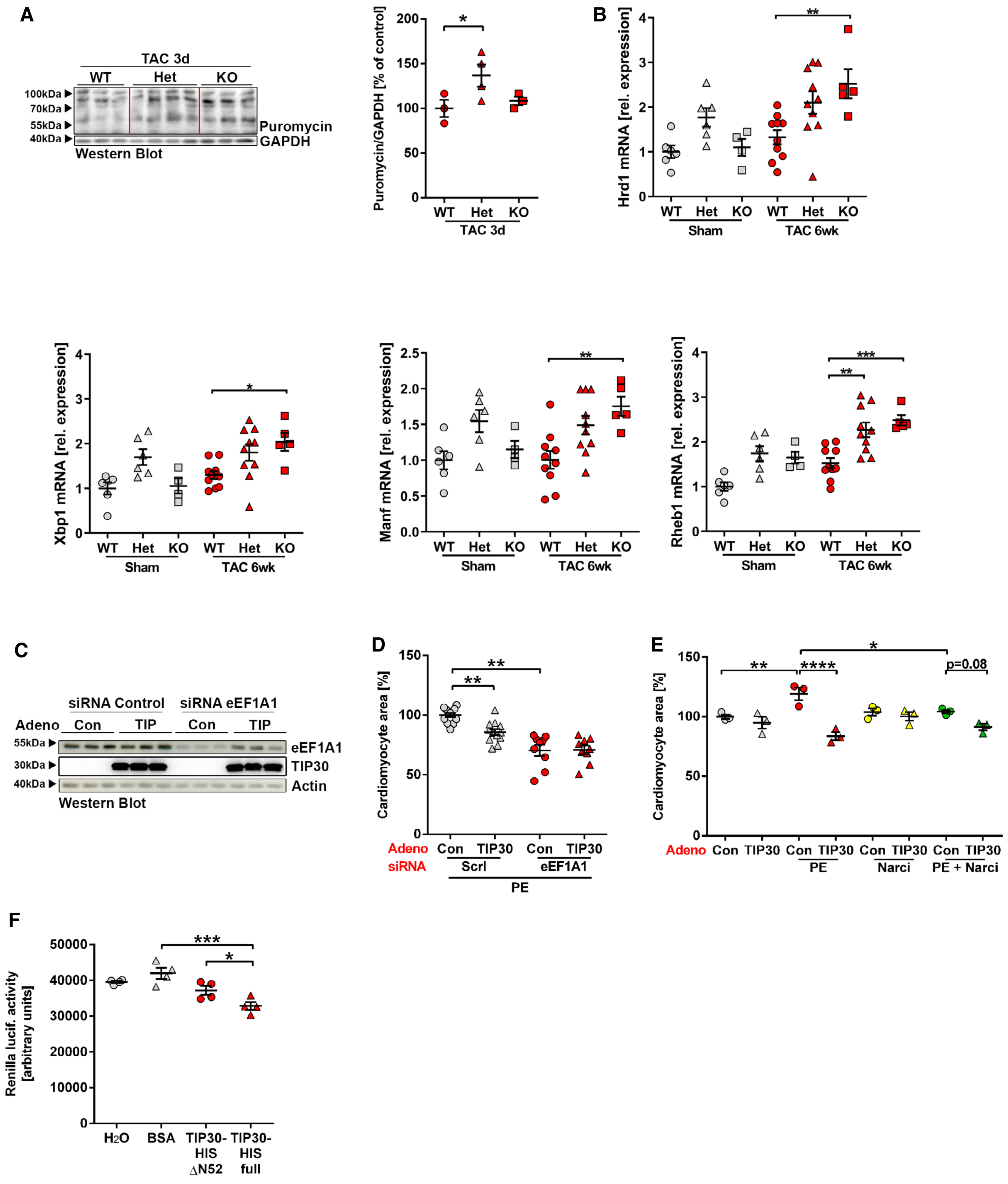
A
TAC 3d
WT Het KO
100kDa
70kDa
55kDa
40kDa
Puromycin
GAPDH
Western Blot
Puromycin/GAPDH [% of control]
WT Het KO
TAC 3d
B
Hrd1 mRNA [rel. expression]
WT Het KO WT Het KO
Sham TAC 6wk
Xbp1 mRNA [rel. expression]
Manf mRNA [rel. expression]
Rheb1 mRNA [rel. expression]
C
siRNA Control siRNA eEF1A1
Adeno Con TIP Con TIP
55kDa
30kDa
40kDa
eEF1A1
TIP30
Actin
Western Blot
D
Cardiomyocyte area [%]
Adeno Con TIP30 Con TIP30
siRNA Scrl eEF1A1
PE
E
Cardiomyocyte area [%]
p=0.08
Adeno Con TIP30 Con TIP30 Con TIP30 Con TIP30
PE Narci PE + Narci
F
Renilla lucif. activity [arbitrary units]
H2O BSA TIP30-HIS ΔN52 TIP30-HIS full

Figure EV5.

Figure EV5. TIP30 inhibits cardiomyocyte protein synthesis by inhibiting eEF1A.

A Western blot analysis of puromycin incorporation in hearts 3 days after TAC surgery in TIP30 WT, Het, and KO mice and their quantification (N = 3–4 mice/group). Puromycin was injected 3 h prior to sacrifice. *$P < 0.05$. One-way ANOVA with Sidak's multiple comparisons test.

B Quantification of Hrd1, Xbp1, Manf, and Rheb1 mRNA abundance by qPCR (N = 4–10 mice/group). *$P < 0.05$, **$P < 0.01$, ***$P < 0.001$. One-way ANOVA with Sidak's multiple comparisons test.

C Western blot of eEF1A1, TIP30, and Actin in NRCM after adenoviral transduction with control virus (Ad.Con) or Ad.TIP30 and transfection with siRNA against eEF1A1 (siRNA eEF1A1) or control siRNA (siRNA control) and treated with phenylephrine (PE) as indicated.

D Cardiomyocyte area in conditions as described in (C) (N = 9–12 samples/group). **$P < 0.01$. One-way ANOVA with Sidak's multiple comparisons test.

E Cell size of NRCM at baseline (Control) or after stimulation with phenylephrine (PE) and narciclasine (Narci) for 24 h as indicated (N = 3 samples/group). *$P < 0.05$, **$P < 0.01$, ****$P < 0.0001$. One-way ANOVA with Sidak's multiple comparisons test.

F Renilla luciferase activity in an *in vitro* translation rabbit reticulocyte lysate system. Bovine serum albumin (BSA) as control, purified TIP30-His full-length protein (TIP30 full) or TIP30-ΔN52-His, was added to the system as indicated (N = 4 replicates/group). *$P < 0.05$, ***$P < 0.001$. One-way ANOVA with Sidak's multiple comparisons test.

Data information: Data are shown as mean ± SEM.
Source data are available online for this figure.

© 2019 The Authors
