# Supplementary material for: TIP30 counteracts cardiac hypertrophy and failure by inhibiting translational elongation
Source: EMBO Mol Med. 2019 Aug 30;11(10):e10018. doi: 10.15252/emmm.201810018 (PMC6783653; doi:10.15252/emmm.201810018)
Supplement: Supplementary file 3 — Source Data for Expanded View and Appendix [file EMMM-11-e10018-s009.zip › Source_Data_for_EV_Figures.pdf]

Source data to EV Figure 1H

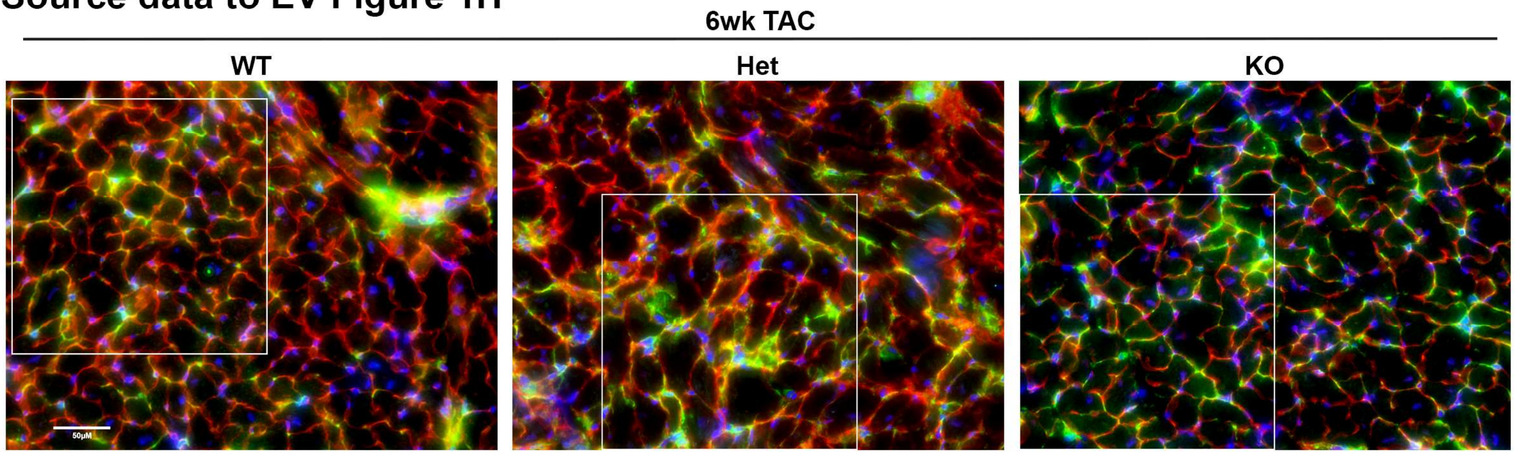

Full microscopy images of heart sections with rectangels depicting the areas shown in EV Fig. 1H (green - PDGFRα, red - WGA, blue - DAPI)

Source data to EV Figure 1K

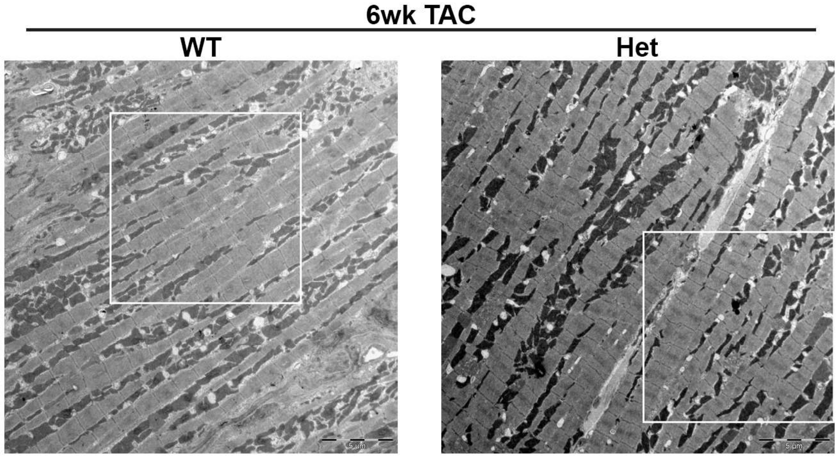

Full electron microscopy images of heart sections with rectangels depicting the areas shown in EV Fig. 1K

Source data to EV Figure 1L

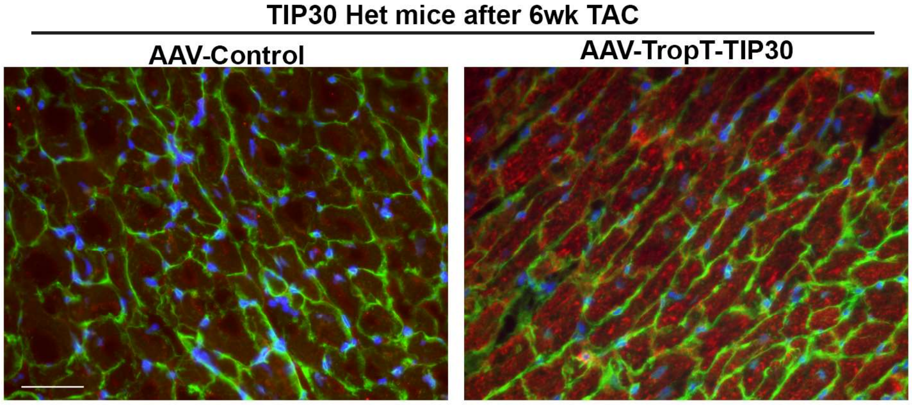

Full microscopy images of heart sections shown in EV Fig. 1L (green - WGA, red - TIP30, blue - DAPI)

Source data to EV Figure 1M

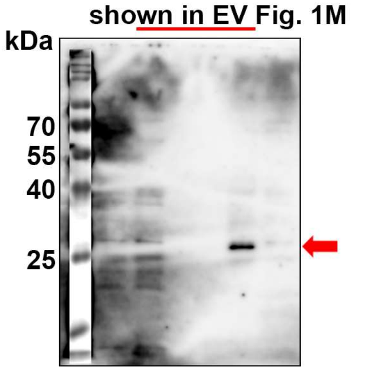

Full unedited Western Blot membrane incubated with anti-TIP30 (Abcam #ab177961)

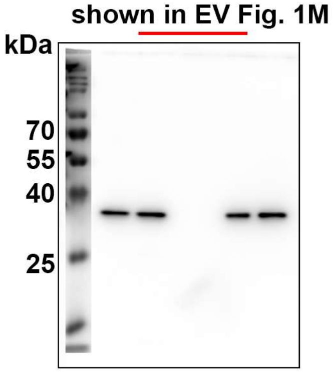

Full unedited Western Blot membrane stripped and incubated with anti-GAPDH (Fitzgerald #10R-G109a)

Source data to EV Figure 2F

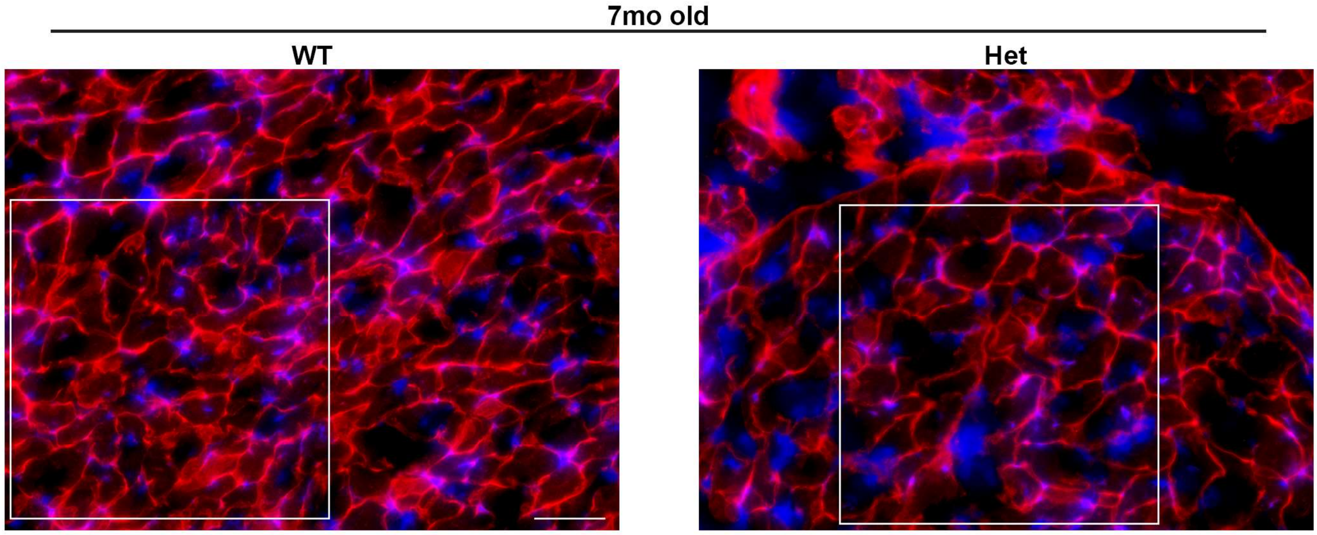

Full microscopy images of heart sections with rectangels depicting the areas shown in EV Fig. 2F (red - WGA, blue - DAPI)

Source data to EV Figure 3A

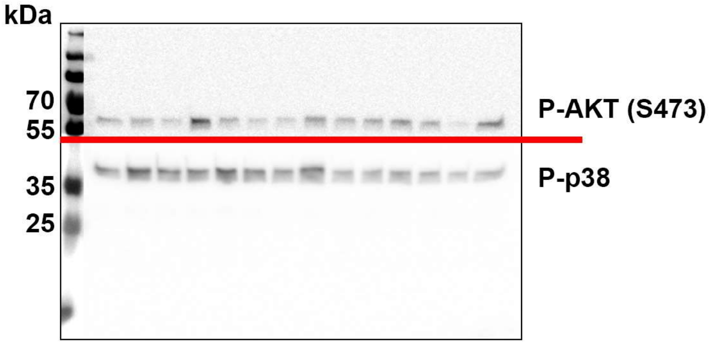

Full unedited Western Blot membrane incubated with anti-Phospho-AKT (S473, Cell Signaling #9271) and anti-Phospho-p38 (Cell Signaling #9211); membrane was cut

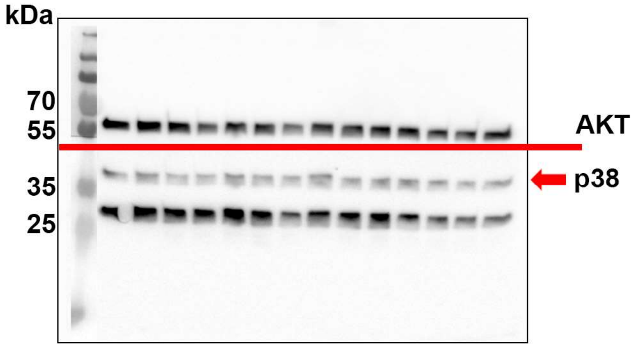

Full unedited Western Blot membrane stripped and incubated with anti-AKT (Cell Signaling #9272) and anti-p38 (Cell Signaling #9212); membrane was cut

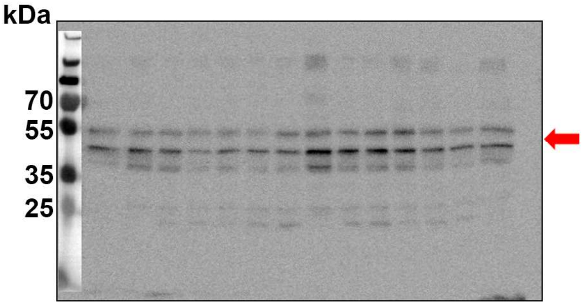

Full unedited Western Blot membrane incubated with anti-Phospho-SAPK/JNK (T183/Y185; Cell Signaling #9251)

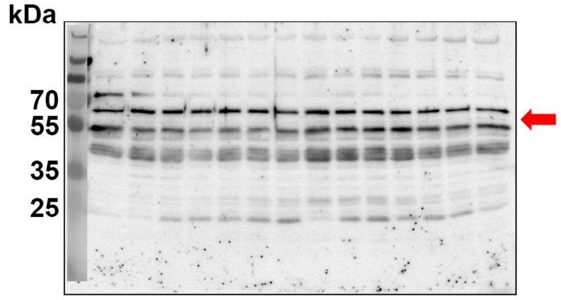

Full unedited Western Blot membrane stripped and incubated with anti-SAPK/JNK (Cell Signaling #9252)

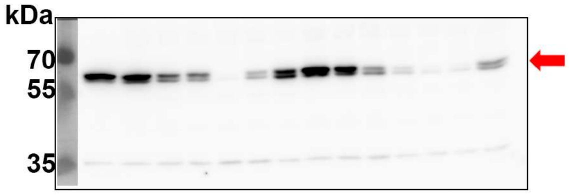

Full unedited Western Blot membrane incubated with anti-Phospho-p70S6 kinase (S371; Cell Signaling #9208); the membrane was cut

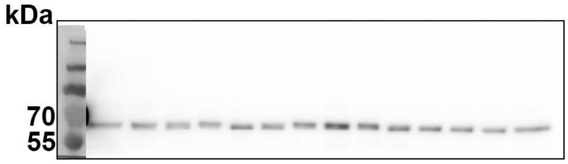

Full unedited Western Blot membrane stripped and incubated with anti-p70S6 kinase (Cell Signaling #2708); the membrane was cut

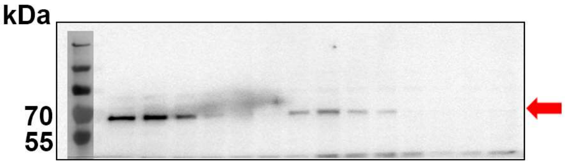

Full unedited Western Blot membrane incubated with anti-Phospho-p70S6 kinase (T389; Cell Signaling #9234); the membrane was cut

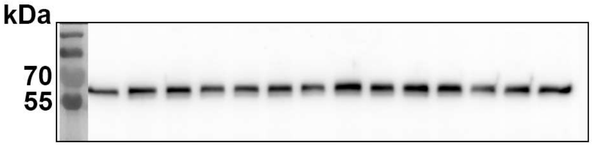

Full unedited Western Blot membrane stripped and incubated with anti-p70S6 kinase (Cell Signaling #2708); the membrane was cut

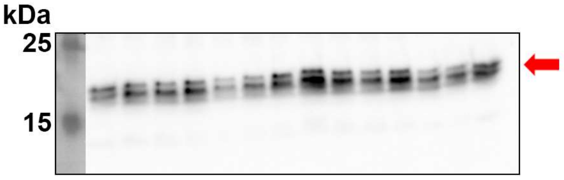

Full unedited Western Blot membrane incubated with anti-Phospho-4E-BP1 (T37/46; Cell Signaling #2855); the membrane was cut

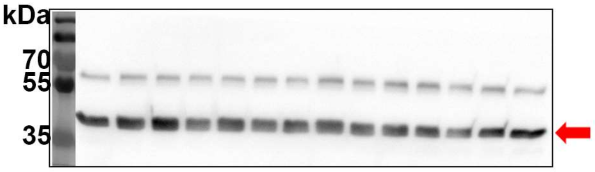

Full unedited Western Blot membrane stripped and incubated with anti-Actin (Sigma #2066); the membrane was cut

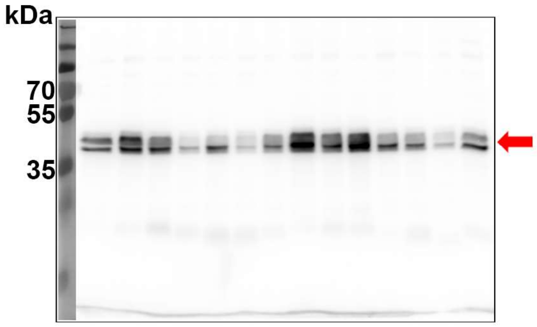

Full unedited Western Blot membrane incubated with anti-Phospho-ERK1/2 (T202/Y204; Cell Signaling #9101)

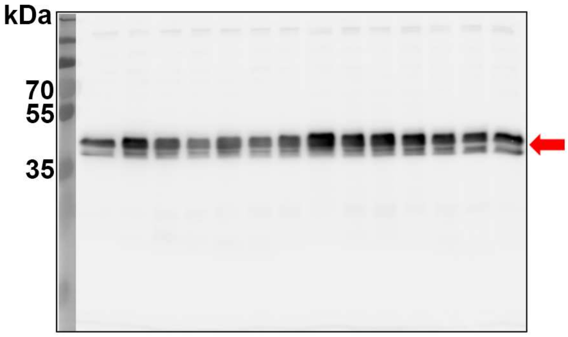

Full unedited Western Blot membrane stripped and incubated with anti-ERK1/2 (Cell Signaling #9102)

Source data to EV Figure 3A

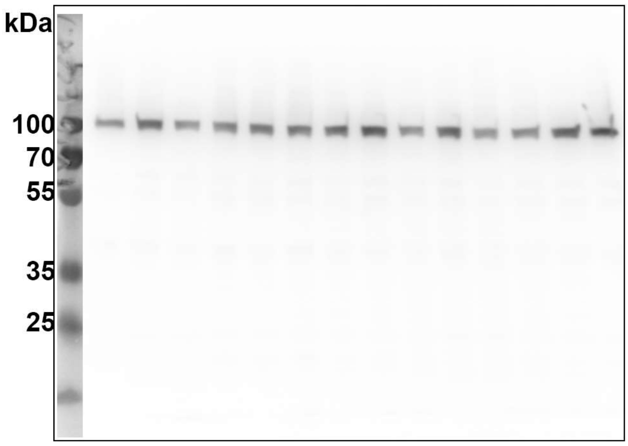

Full unedited Western Blot membrane incubated with anti-Phospho-eEF2 (T56, Cell Signaling #2331)

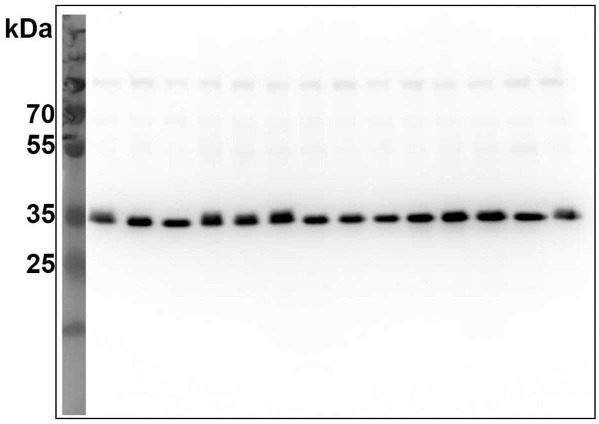

Full unedited Western Blot membrane stripped and incubated with anti-GAPDH (Fitzgerald #10R-G109a)

shown in EV Fig. 3A

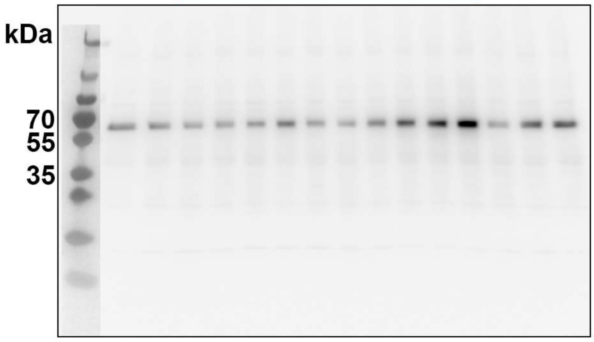

Full unedited Western Blot membrane incubated with anti-Phospho-AMPKα (T172, Cell Signaling #2235)

shown in EV Fig. 3A

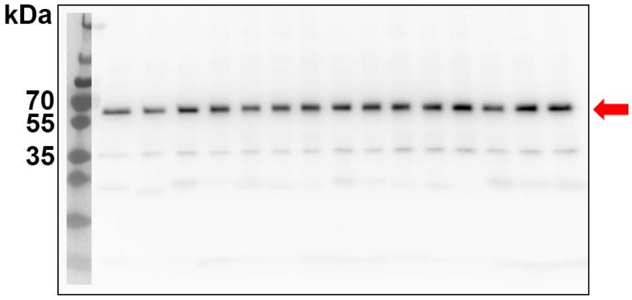

Full unedited Western Blot membrane stripped and incubated with anti-AMPKα (Cell Signaling #2532)

shown in EV Fig. 3A

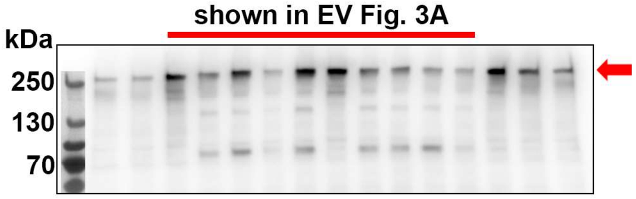

Full unedited Western Blot membrane incubated with anti-Phospho-mTOR (S2448, Cell Signaling #5536); membrane was cut

shown in EV Fig. 3A

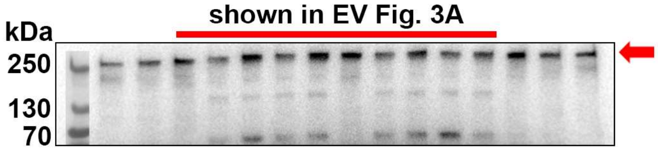

Full unedited Western Blot membrane striped and incubated with anti-mTOR (Cell Signaling #29836); membrane was cut

shown in EV Fig. 3A

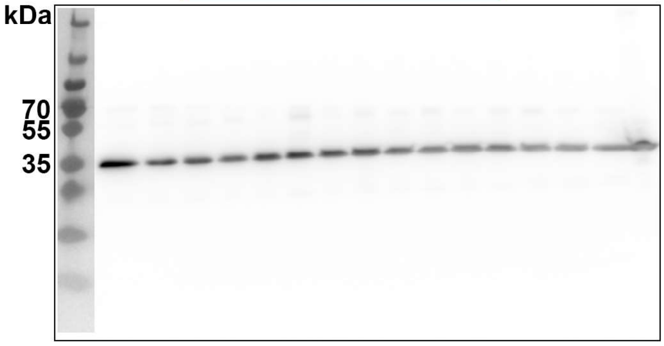

Full unedited Western Blot membrane stripped and incubated with anti-GAPDH (Fitzgerald #10R-G109a)

Source data to EV Figure 4B

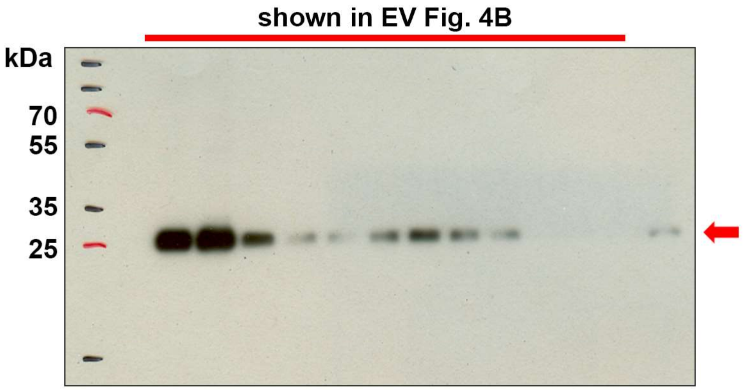

Full unedited Western Blot membrane
incubated with anti-S6 (Cell Signaling #2217)

Source data to EV Figure 4F

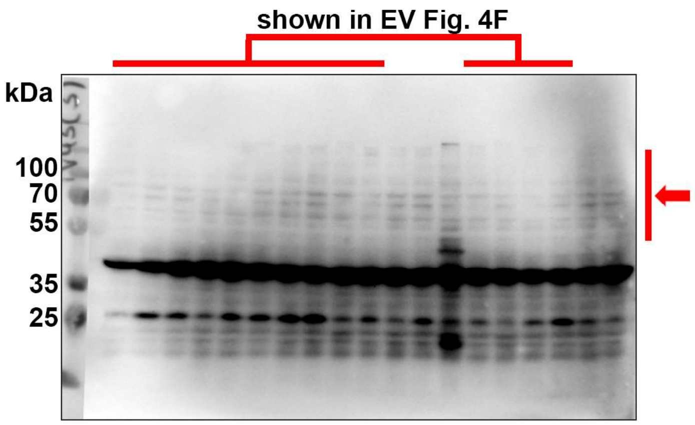

Full unedited Western Blot membrane
incubated with anti-Puromycin (Sigma #MABE341)

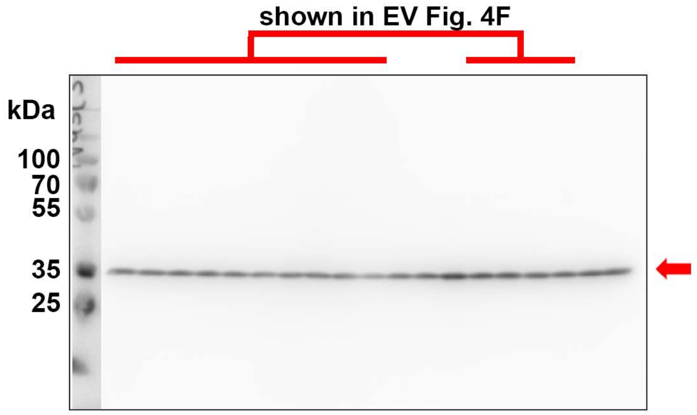

Full unedited Western Blot membrane stripped
and incubated with anti-GAPDH (Fitzgerald #10R-G109a)

Source data to EV Figure 5A

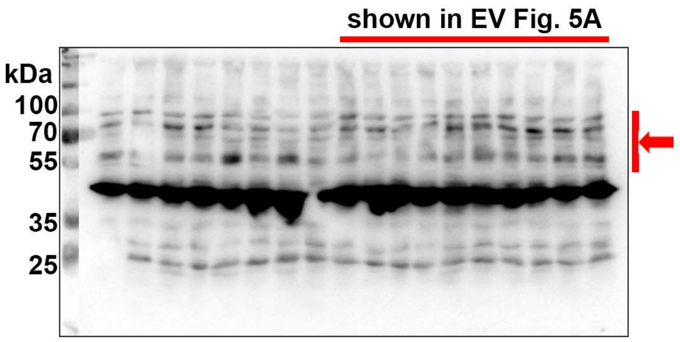

Full unedited Western Blot membrane
incubated with anti-Puromycin (Sigma #MABE341)

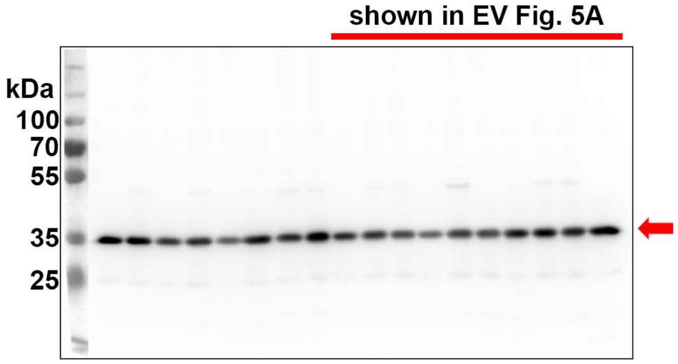

Full unedited Western Blot membrane stripped
and incubated with anti-GAPDH (Fitzgerald #10R-G109a)

Source data to EV Figure 5C

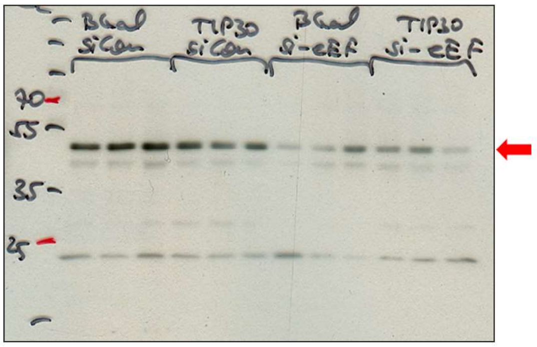

Full unedited Western Blot membrane
incubated with anti-eEF1A1 (Abcam #ab37969)

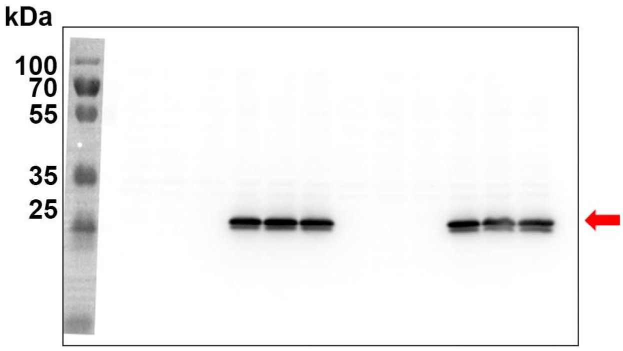

Full unedited Western Blot membrane
incubated with anti-TIP30 (Abcam #ab177961)

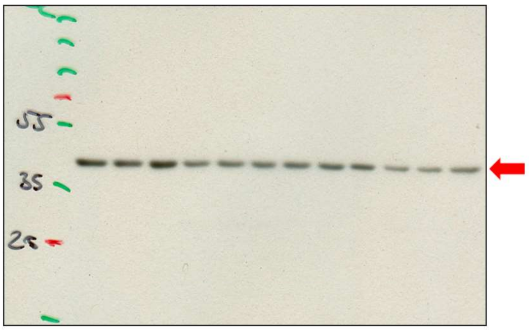

Full unedited Western Blot membrane stripped and
incubated with anti-Actin (Sigma #2066)
