# Supplementary material for: TIP30 counteracts cardiac hypertrophy and failure by inhibiting translational elongation
Source: EMBO Mol Med. 2019 Aug 30;11(10):e10018. doi: 10.15252/emmm.201810018 (PMC6783653; doi:10.15252/emmm.201810018)
Supplement: Supplementary file 3 — Source Data for Expanded View and Appendix [file EMMM-11-e10018-s009.zip › Source_Data_for_Appendix_Figures.pdf]

Source data to Appendix Figure S1A

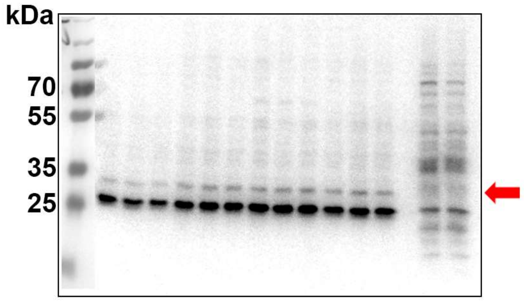

Full unedited Western Blot membrane incubated with anti-TIP30 (Abcam #ab177961)

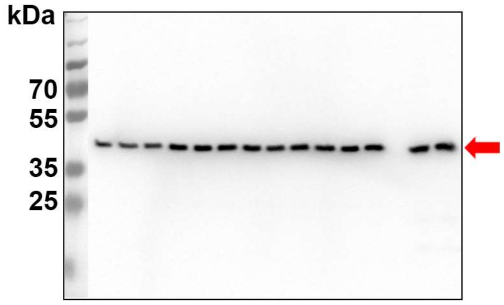

Full unedited Western Blot membrane stripped and incubated with anti-Actin (Sigma #2066)

Source data to Appendix Figure S1B

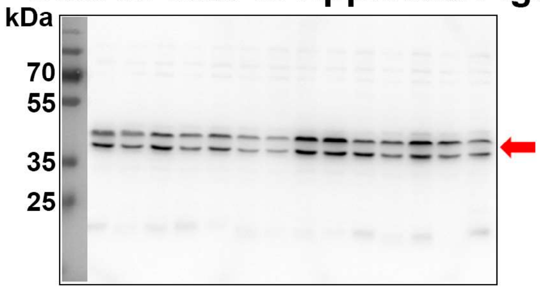

Full unedited Western Blot membrane incubated with anti-Phospho-ERK1/2 (T202/Y204; Cell Signaling #9101)

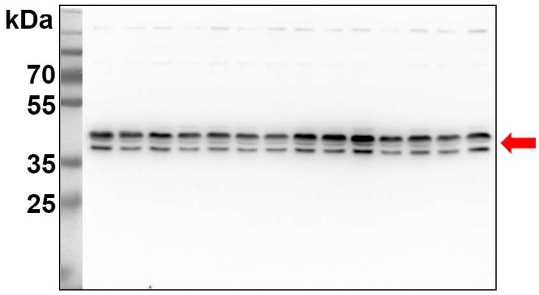

Full unedited Western Blot membrane stripped and incubated with anti-ERK1/2 (Cell Signaling #9102)

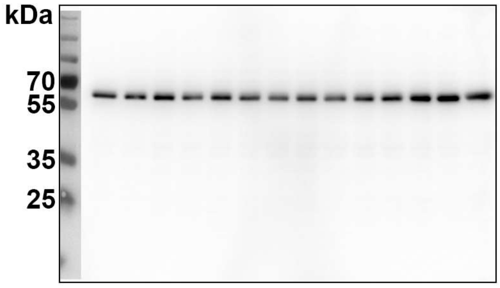

Full unedited Western Blot membrane incubated with anti-Phospho-AMPKα (T172, Cell Signaling #2235)

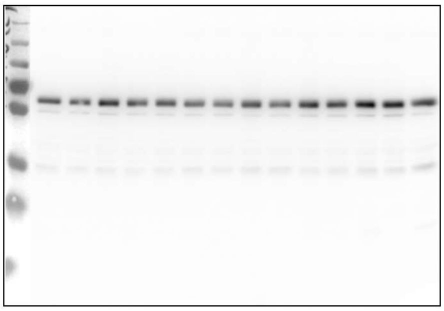

Full unedited Western Blot membrane stripped and incubated with anti-AMPKα (Cell Signaling #2532)

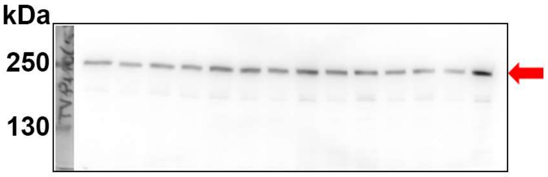

Full unedited Western Blot membrane incubated with anti-Phospho-mTOR (S2448, Cell Signaling #5536); membrane was cut

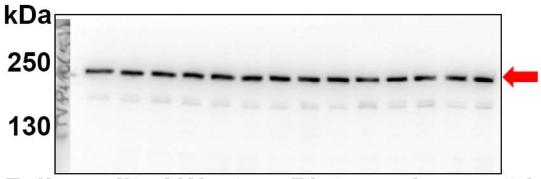

Full unedited Western Blot membrane striped and incubated with anti-mTOR (Cell Signaling #29836); membrane was cut

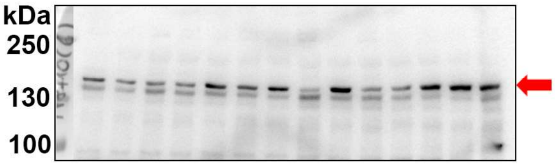

Full unedited Western Blot membrane incubated with anti-Phospho-Raptor (S792, Cell Signaling #2083); membrane was cut

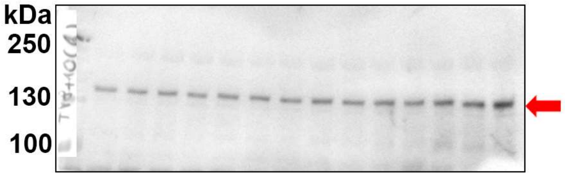

Full unedited Western Blot membrane incubated with anti-Raptor (Cell Signaling #2280); membrane was cut

Source data to Appendix Figure S1B

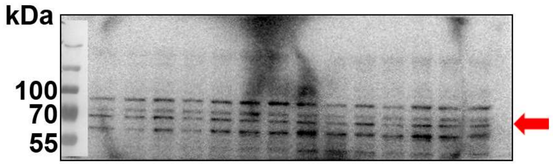

Full unedited Western Blot membrane incubated with anti-Phospho-p70S6 kinase (S371; Cell Signaling #9208); the membrane was cut

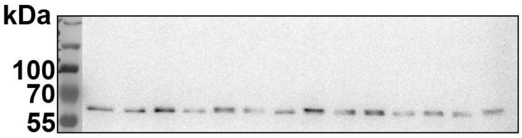

Full unedited Western Blot membrane stripped and incubated with anti-p70S6 kinase (Cell Signaling #2708); the membrane was cut

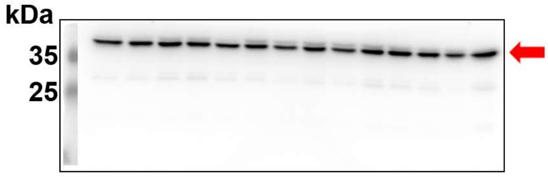

Full unedited Western Blot membrane incubated with anti-Phospho-p38 (Cell Signaling #9211); membrane was cut

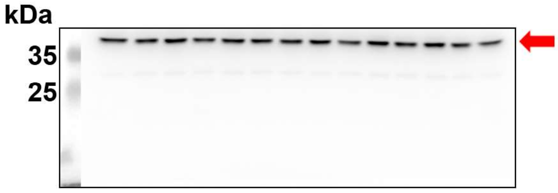

Full unedited Western Blot membrane stripped and incubated with anti-p38 (Cell Signaling #9212); membrane was cut

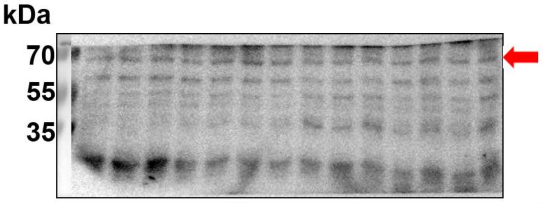

Full unedited Western Blot membrane incubated with anti-Phospho-p70S6 kinase (T389; Cell Signaling #9234); the membrane was cut

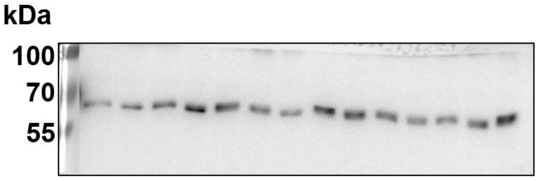

Full unedited Western Blot membrane stripped and incubated with anti-p70S6 kinase (Cell Signaling #2708); the membrane was cut

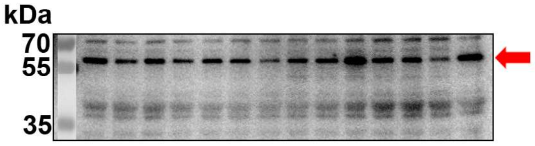

Full unedited Western Blot membrane incubated with anti-Phospho-AKT (S473, Cell Signaling #9271); membrane was cut

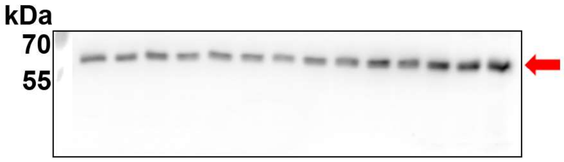

Full unedited Western Blot membrane stripped and incubated with anti-AKT (Cell Signaling #9272); membrane was cut

Source data to Appendix Figure S1C

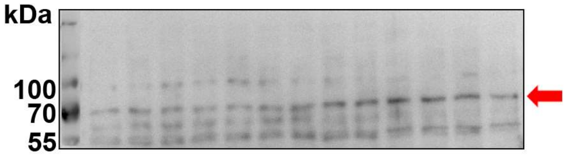

Full unedited Western Blot membrane incubated with anti-Phospho-p70S6 kinase (S371; Cell Signaling #9208); the membrane was cut

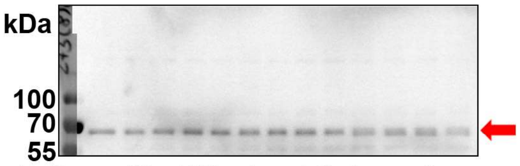

Full unedited Western Blot membrane stripped and incubated with anti-p70S6 kinase (Cell Signaling #2708); the membrane was cut

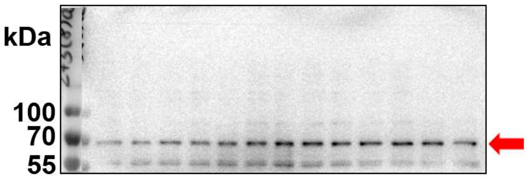

Full unedited Western Blot membrane incubated with anti-Phospho-p70S6 kinase (T389; Cell Signaling #9234); the membrane was cut

Source data to Appendix Figure S1C

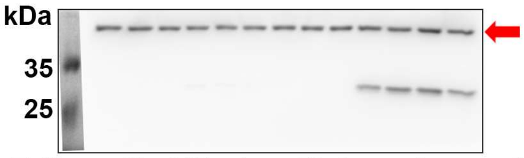

Full unedited Western Blot membrane stripped
and incubated with anti-Actin
(Sigma #2066); the membrane was cut

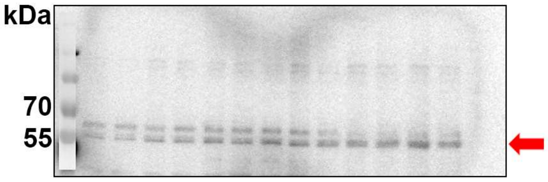

Full unedited Western Blot membrane incubated
with anti-Phospho-AKT (S473, Cell Signaling #9271);
membrane was cut

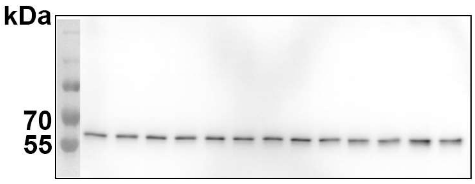

Full unedited Western Blot membrane stripped
and incubated with anti-AKT (Cell Signaling #9272);
membrane was cut

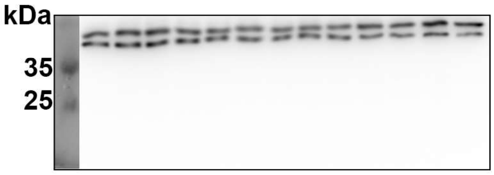

Full unedited Western Blot membrane stripped
and incubated with anti-Phospho-ERK1/2
(Cell Signaling #9101); the membrane was cut

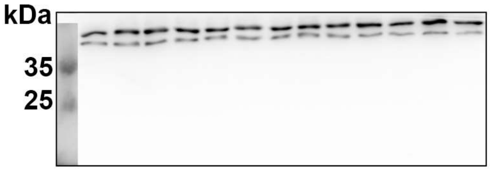

Full unedited Western Blot membrane stripped
and incubated with anti-ERK1/2
(Cell Signaling #9102); the membrane was cut

Source data to Appendix Figure S3A

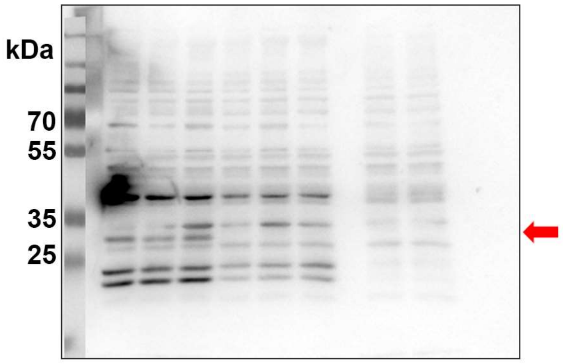

Full unedited Western Blot membrane
incubated with anti-TIP30 (Abcam #ab177961)

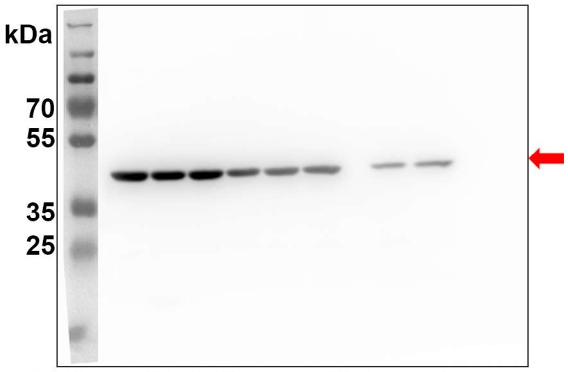

Full unedited Western Blot membrane
stripped and incubated with anti-Actin (Sigma #2066)
