# Supplementary material for: TIP30 counteracts cardiac hypertrophy and failure by inhibiting translational elongation
Source: EMBO Mol Med. 2019 Aug 30;11(10):e10018. doi: 10.15252/emmm.201810018 (PMC6783653; doi:10.15252/emmm.201810018)
Supplement: Supplementary file 5 — Source Data for Figure 1 [file EMMM-11-e10018-s003.pdf]

Source data to Figure 1A

shown in Fig. 1A

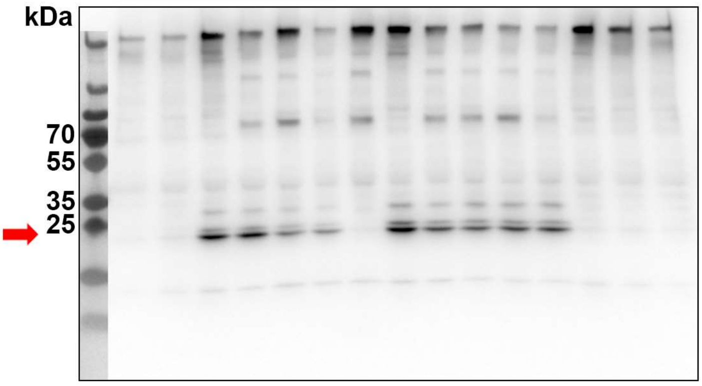

Full unedited Western Blot membrane incubated with anti-TIP30 (Abcam #ab177961)

shown in Fig. 1A

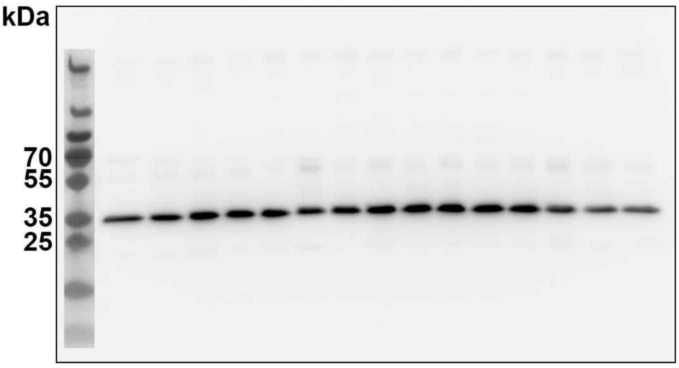

Full unedited Western Blot membrane stripped and incubated with anti-GAPDH (Fitzgerald #10R-G109a)

Source data to Figure 1M

WT TAC 6wk

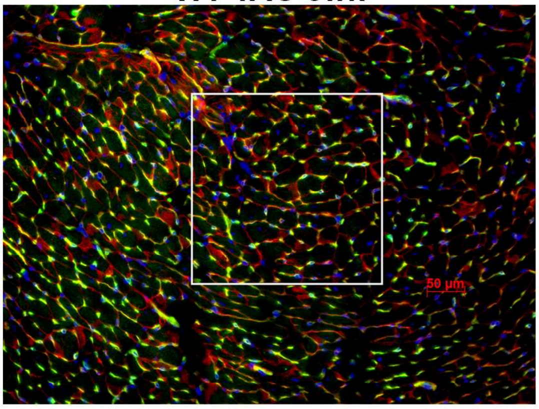

Het TAC 6wk

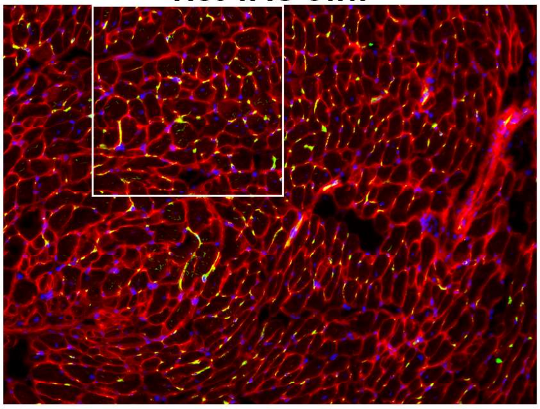

Full microscopy images of heart sections with rectangels depicting the areas shown in Fig. 1M (green - Isolectin, red - WGA, blue - DAPI)

Source data to Figure 1P

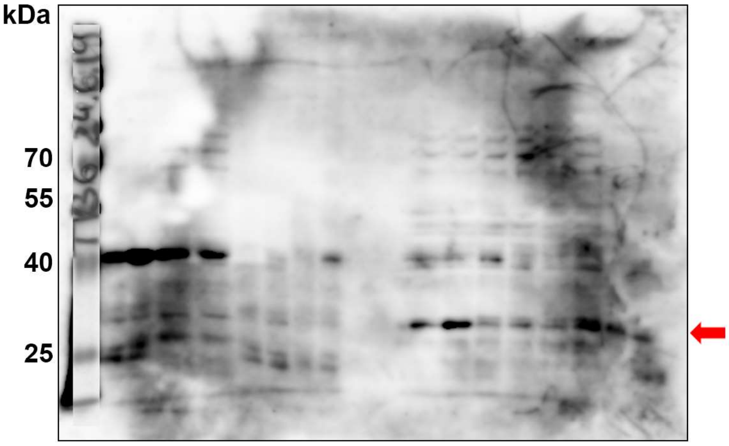

Full unedited Western Blot membrane incubated with anti-TIP30 (Abcam #ab177961)

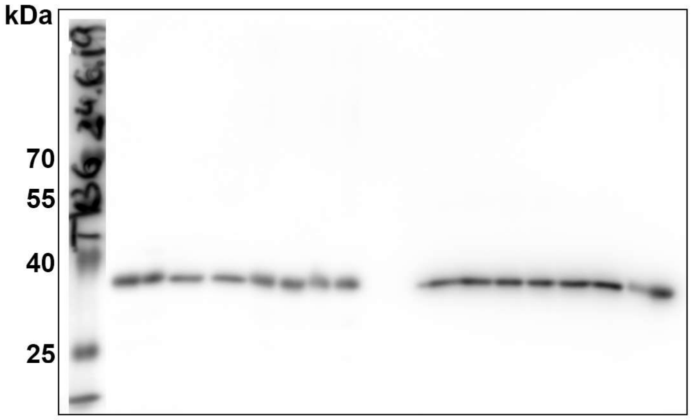

Full unedited Western Blot membrane stripped and incubated with anti-GAPDH (Fitzgerald #10R-G109a)
