# Supplementary material for: TIP30 counteracts cardiac hypertrophy and failure by inhibiting translational elongation
Source: EMBO Mol Med. 2019 Aug 30;11(10):e10018. doi: 10.15252/emmm.201810018 (PMC6783653; doi:10.15252/emmm.201810018)
Supplement: Supplementary file 6 — Source Data for Figure 2 [file EMMM-11-e10018-s004.pdf]

Source data to Figure 2A

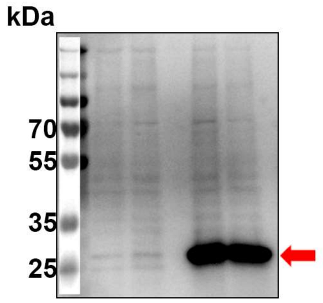

Full unedited Western Blot
membrane incubated with
anti-TIP30 (Abcam #ab177961)

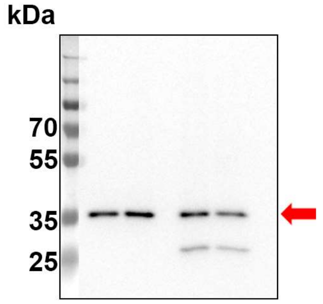

Full unedited Western Blot
membrane stripped and incubated
with anti-GAPDH (Fitzgerald #10R-G109a)

Source data to Figure 2G

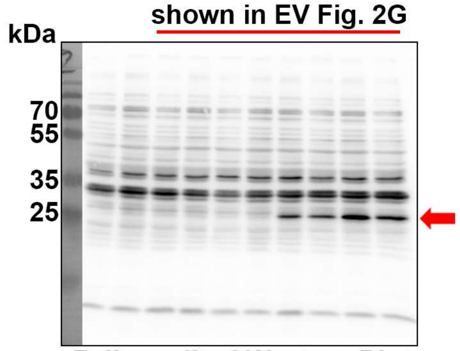

Full unedited Western Blot
membrane incubated with
anti-TIP30 (Abcam #ab177961)

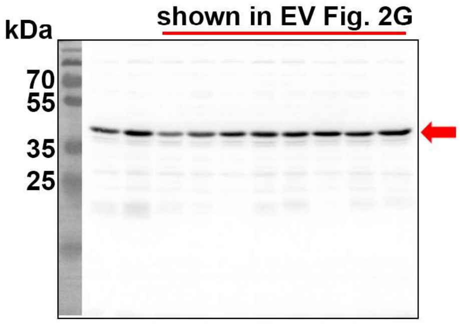

Full unedited Western Blot
membrane stripped and incubated
with anti-Actin (Sigma #2066)
