# Supplementary material for: TIP30 counteracts cardiac hypertrophy and failure by inhibiting translational elongation
Source: EMBO Mol Med. 2019 Aug 30;11(10):e10018. doi: 10.15252/emmm.201810018 (PMC6783653; doi:10.15252/emmm.201810018)
Supplement: Supplementary file 7 — Source Data for Figure 3 [file EMMM-11-e10018-s005.pdf]

Source data to Figure 3A

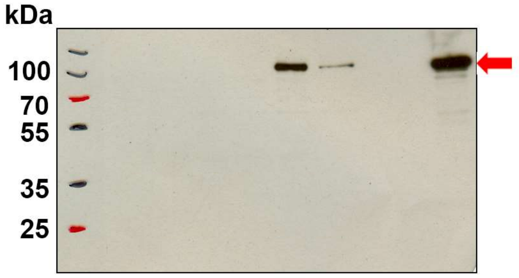

Full unedited Western Blot membrane incubated with anti-Myc (Cell Signaling #2276) to detect Nucleolin-Myc

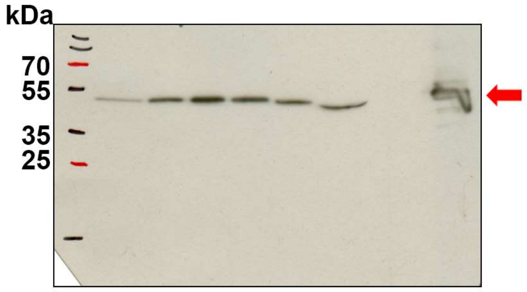

Full unedited Western Blot membrane incubated with anti-Myc (Cell Signaling #2276) to detect eEF1A1-Myc

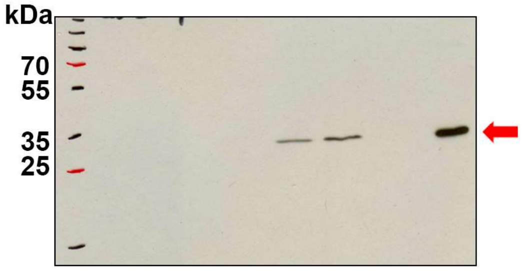

Full unedited Western Blot membrane incubated with anti-Myc (Cell Signaling #2276) to detect Rps3a-Myc

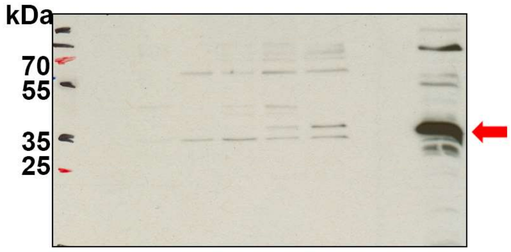

Full unedited Western Blot membrane incubated with anti-Myc (Cell Signaling #2276) to detect hnRNP A2/B1-Myc

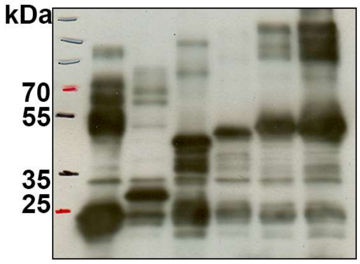

Full unedited Western Blot membrane incubated with anti-GST (Cell Signaling #2625) to detect GST-TIP30

Source data to Figure 3B

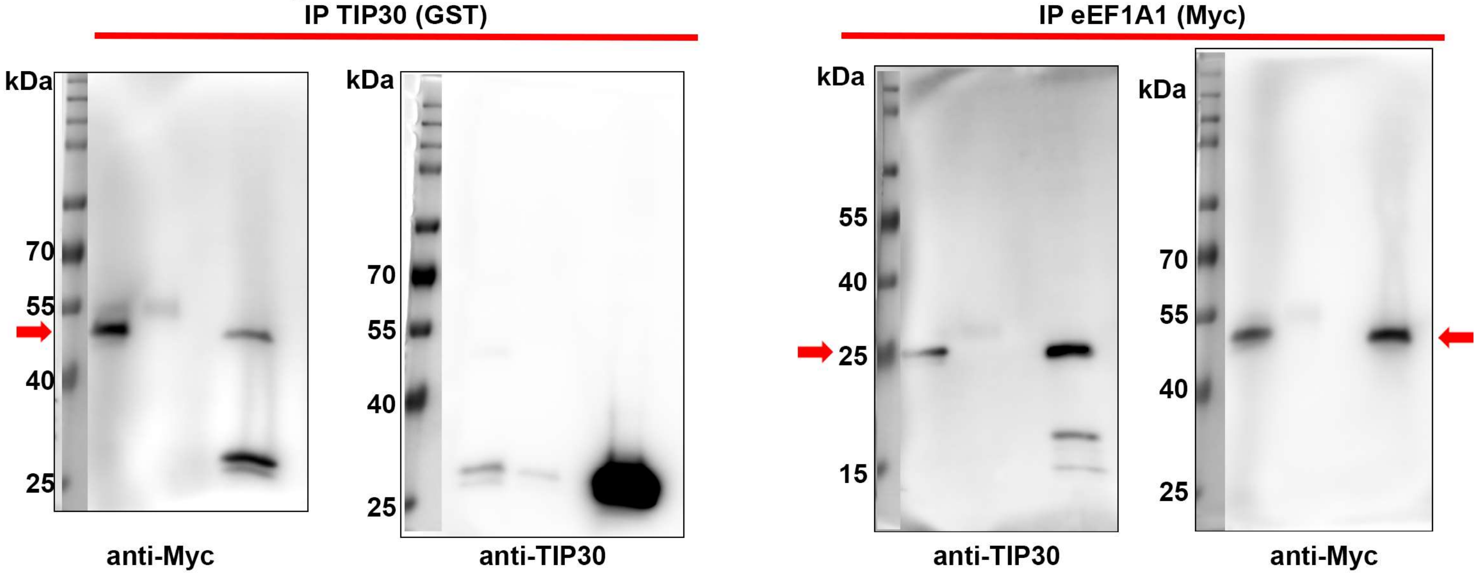

Full unedited Western Blot membranes incubated either with anti-Myc to detect eEF1A1-Myc (Cell Signaling #2276) or anti-TIP30 (Abcam #ab177961)

Source data to Figure 3E

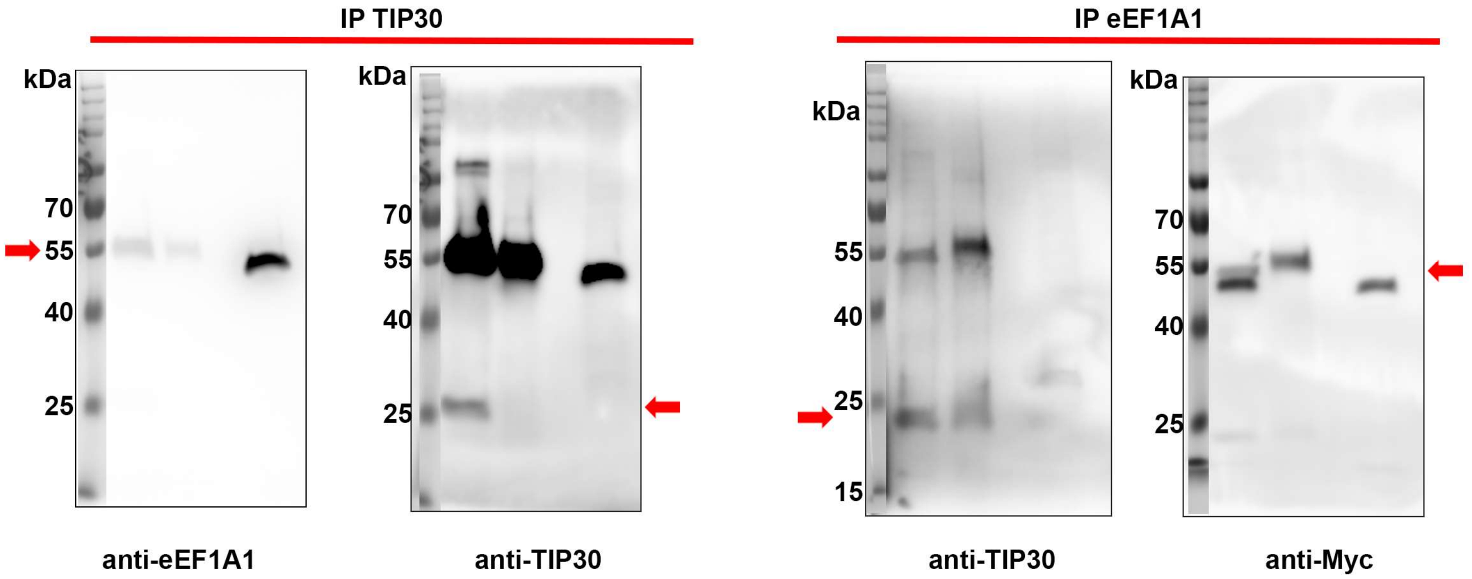

Full unedited Western Blot membranes incubated either with anti-eEF1A1 (Abcam #ab118703) or anti-TIP30 (Abcam #ab177961)

Source data to Figure 3G

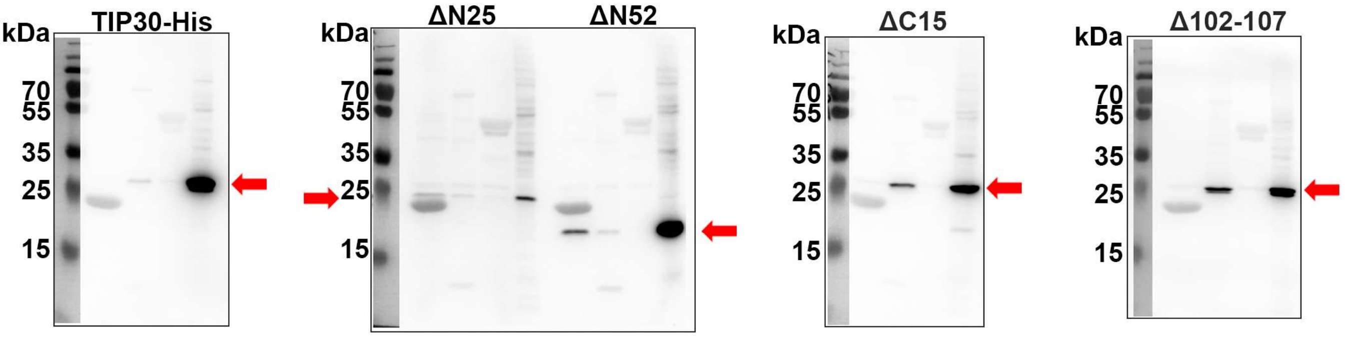

Full unedited Western Blot membranes incubated with anti-His (Cell Signaling #2365)

Source data to Figure 3H

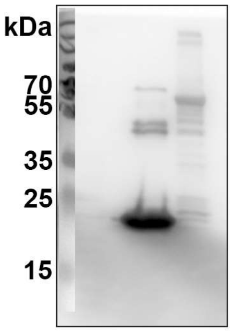

Full unedited Western Blot membranes incubated with anti-GST (Cell Signaling #2625)

Source data to Figure 3I

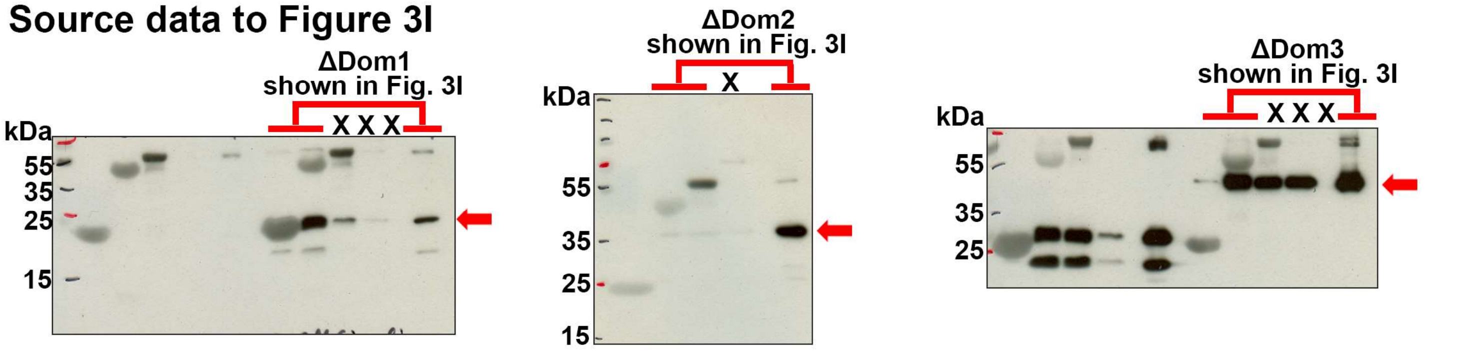

Full unedited Western Blot membranes incubated with anti-His (Cell Signaling #2365)

Source data to Figure 3K

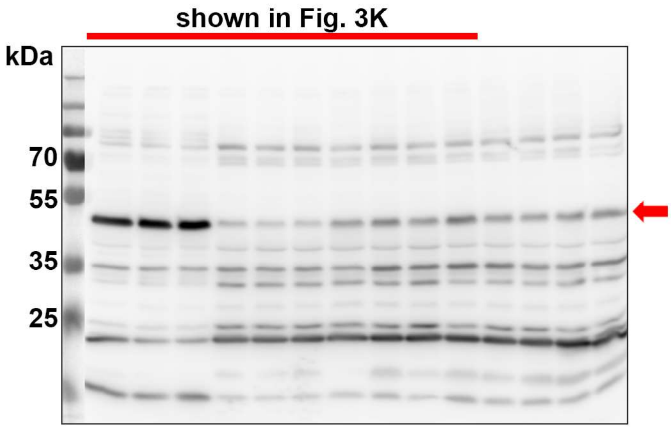

Full unedited Western Blot membrane incubated with anti-eEF1A1 (Abcam #ab118703)

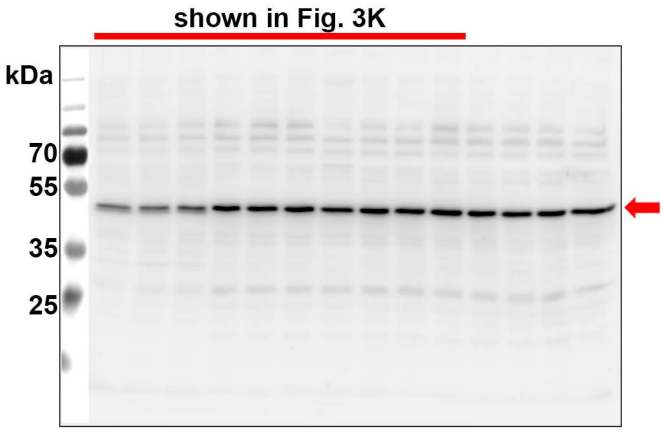

Full unedited Western Blot membrane incubated with anti-eEF1A2 (Abcam #ab156954)

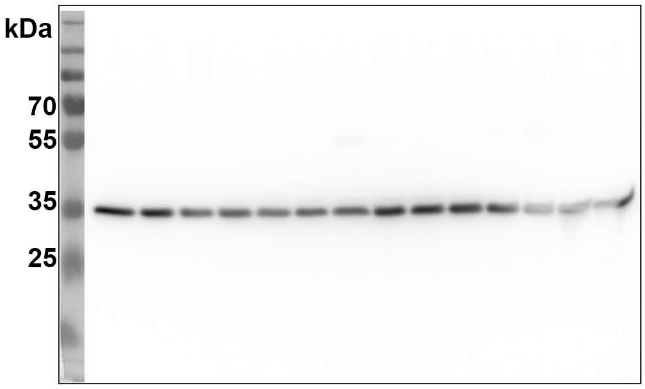

Full unedited Western Blot membrane incubated with anti-GAPDH (Fitzgerald #10R-G109a)

Source data to Figure 3L

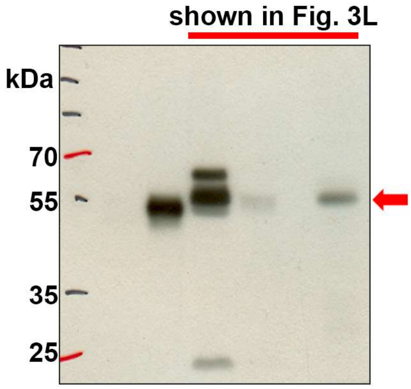

Full unedited Western Blot membrane incubated with anti-GST (Cell Signaling #2625)

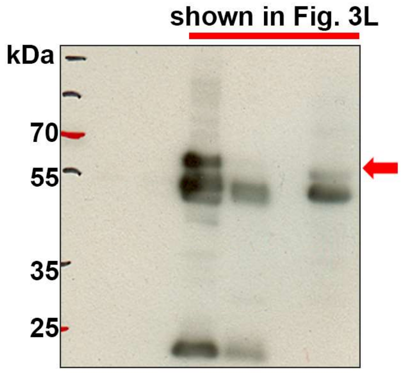

Full unedited Western Blot membrane incubated with anti-HIS (Cell Signaling #2365)
