# Supplementary material for: TIP30 counteracts cardiac hypertrophy and failure by inhibiting translational elongation
Source: EMBO Mol Med. 2019 Aug 30;11(10):e10018. doi: 10.15252/emmm.201810018 (PMC6783653; doi:10.15252/emmm.201810018)
Supplement: Supplementary file 8 — Source Data for Figure 4 [file EMMM-11-e10018-s006.pdf]

Source data to Figure 4A

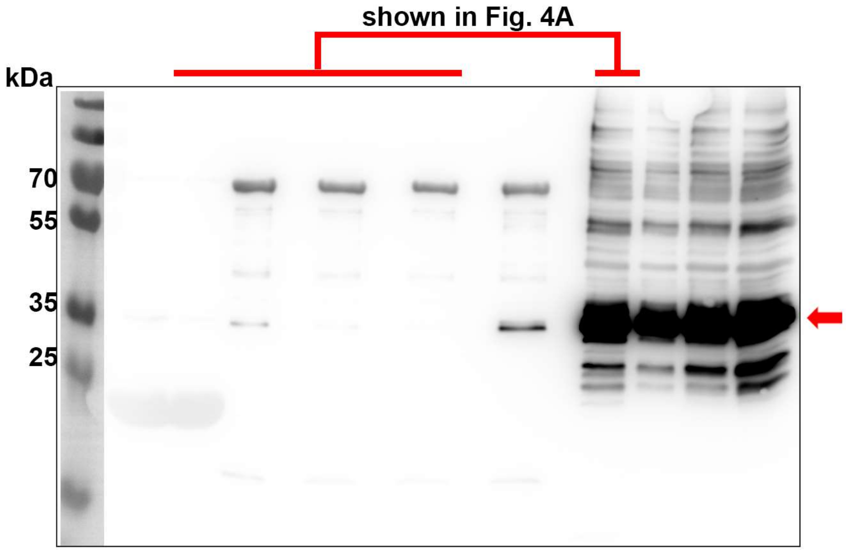

Full unedited Western Blot membrane incubated with anti-eEF1B2 (Abcam #ab77043)

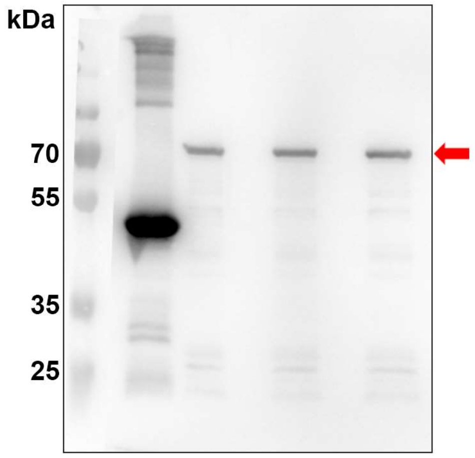

Full unedited Western Blot membrane incubated with anti-GST (Cell Signaling #2625)

Source data to Figure 4B

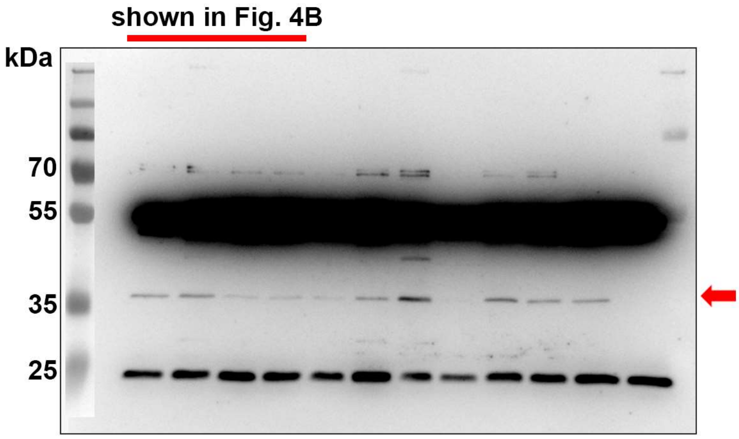

Full unedited Western Blot membrane incubated with anti-eEF1B2 (Abcam #ab77043)

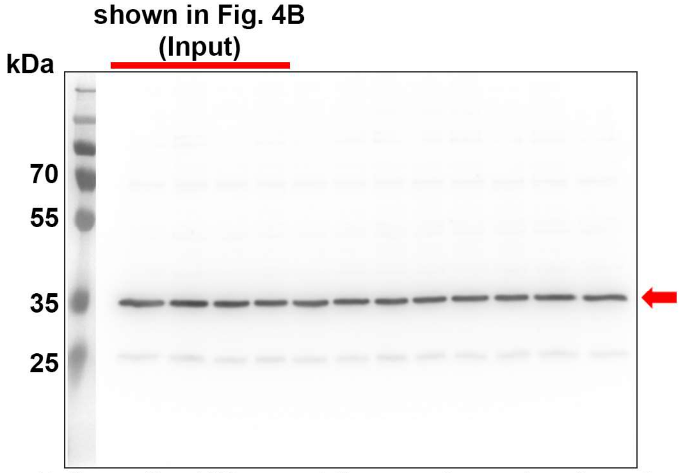

Full unedited Western Blot membrane incubated with anti-eEF1B2 (Abcam #ab77043)

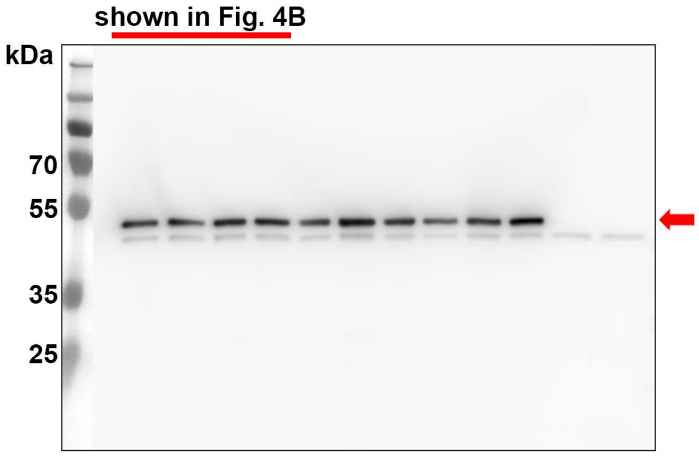

Full unedited Western Blot membrane incubated with anti-Myc (Cell Signaling #2276)

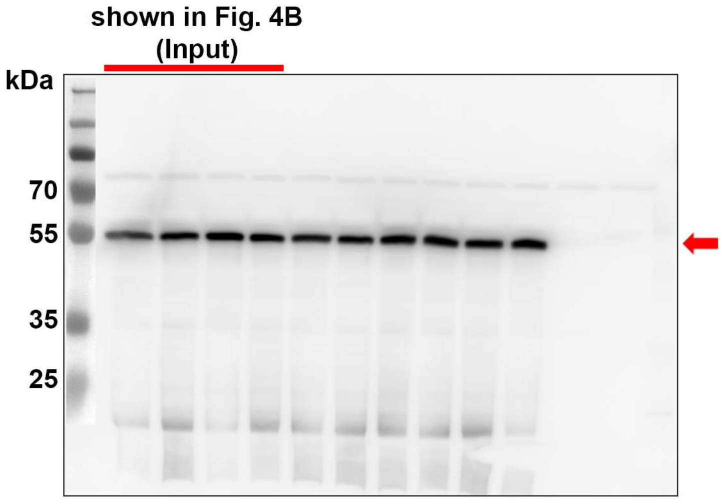

Full unedited Western Blot membrane incubated with anti-Myc (Cell Signaling #2276)

Source data to Figure 4B

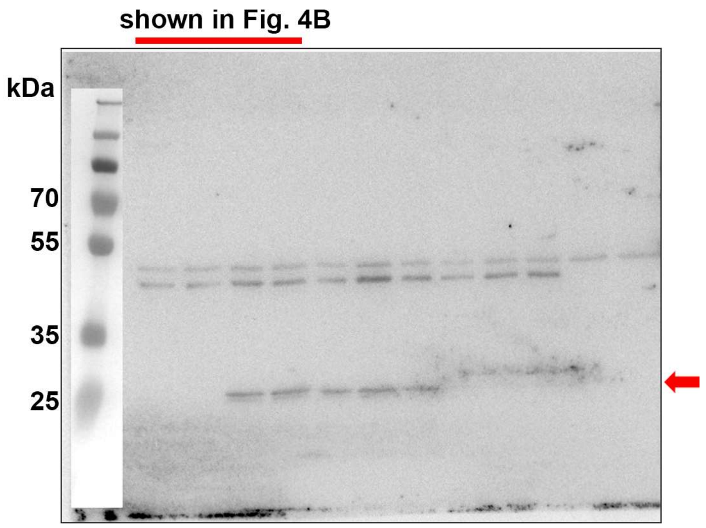

Full unedited Western Blot membrane incubated with anti-TIP30 (Abcam #ab177961)

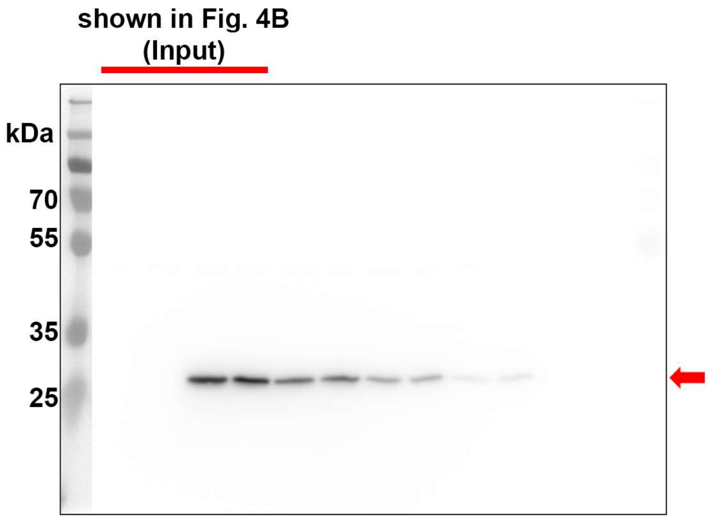

Full unedited Western Blot membrane incubated with anti-TIP30 (Abcam #ab177961)

Source data to Figure 4D

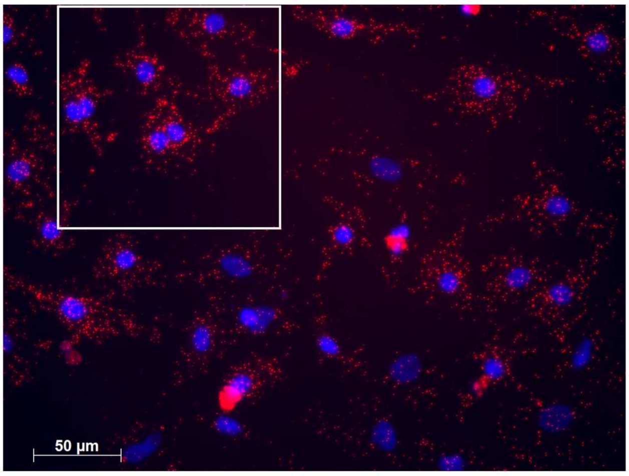

Ad.Con baseline

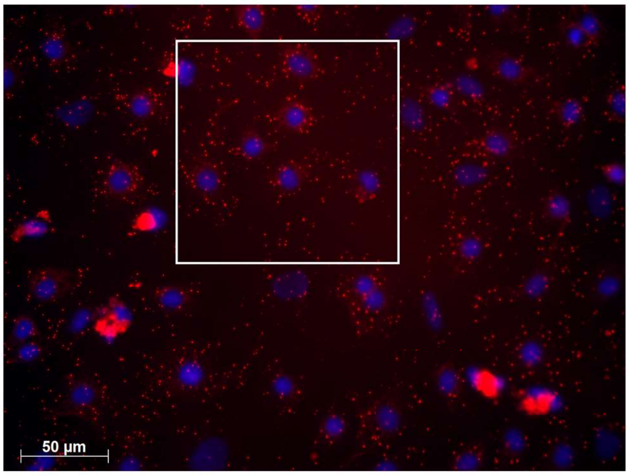

Ad.TIP30 baseline

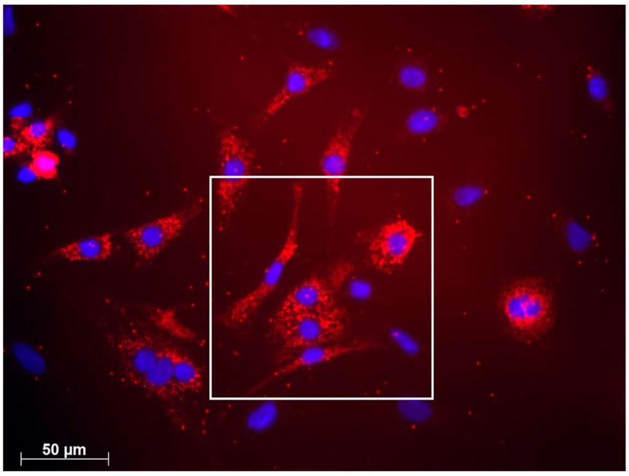

Ad.Con PE

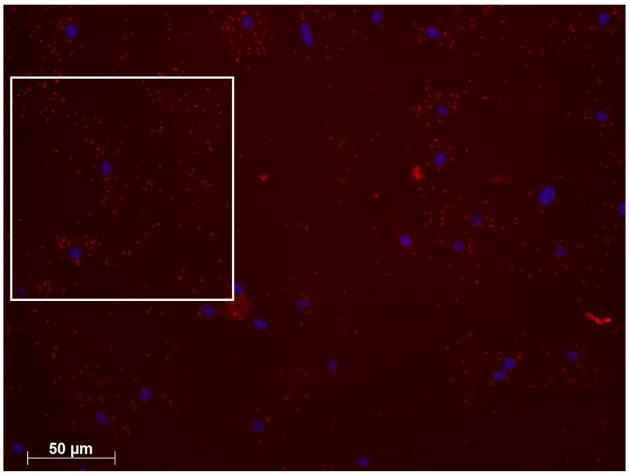

Ad.TIP30 PE

Full microscopy images of NRCM with rectangels depicting the areas shown in Fig. 4D (red - PLA, blue - DAPI)

Source data to Figure 4F

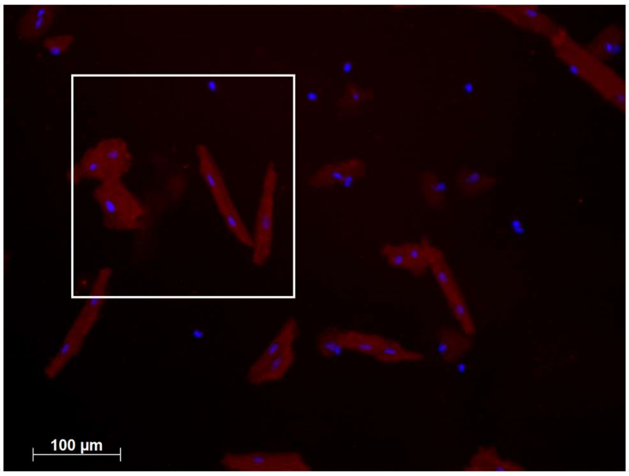

WT Sham

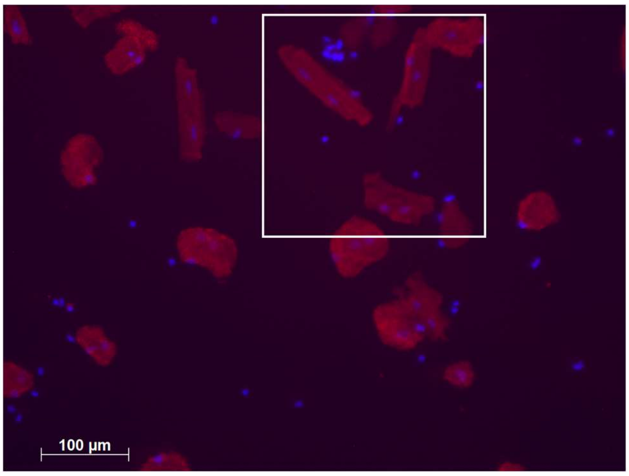

Het Sham

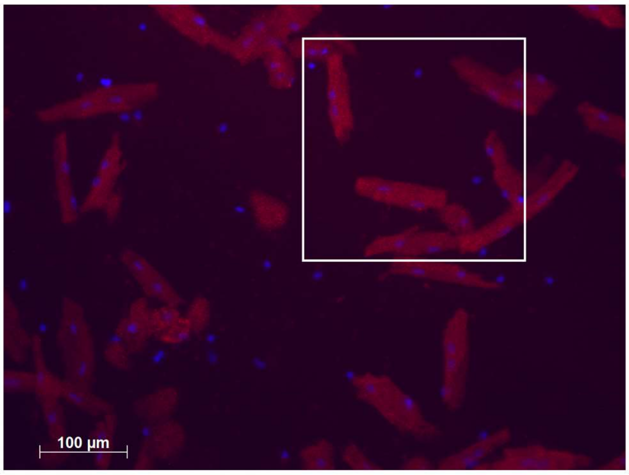

WT TAC

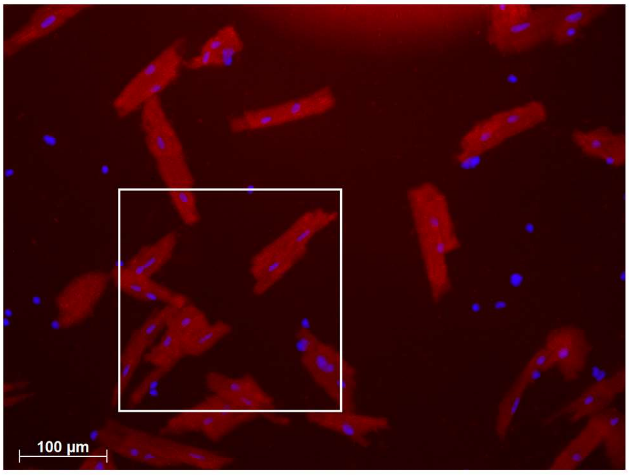

Het TAC

Full microscopy images of isolated adult myocytes with rectangels depicting the areas shown in Fig. 4F (red - PLA, blue - DAPI)
