# Supplementary material for: TIP30 counteracts cardiac hypertrophy and failure by inhibiting translational elongation
Source: EMBO Mol Med. 2019 Aug 30;11(10):e10018. doi: 10.15252/emmm.201810018 (PMC6783653; doi:10.15252/emmm.201810018)
Supplement: Supplementary file 9 — Source Data for Figure 5 [file EMMM-11-e10018-s007.pdf]

Source data to Figure 5A

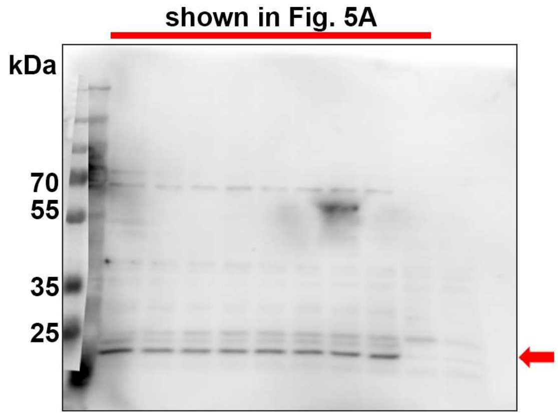

Full unedited Western Blot membrane incubated with anti-TIP30 (Abcam #ab177961)

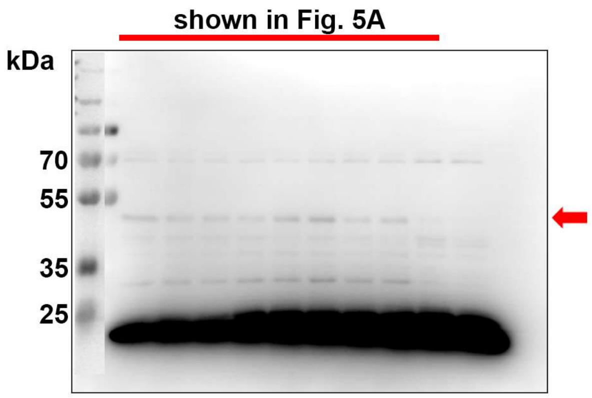

Full unedited Western Blot membrane incubated with anti-eEF1A1 (Abcam #ab118703)

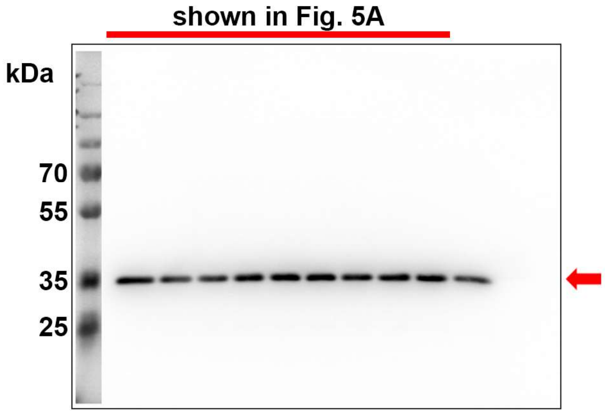

Full unedited Western Blot membrane stripped and incubated with anti-GAPDH (Fitzgerald #10R-G109a)

Source data to Figure 5C

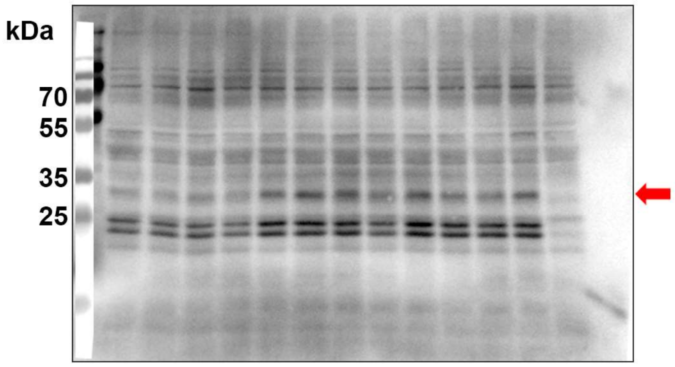

Full unedited Western Blot membrane incubated with anti-TIP30 (Abcam #ab177961)

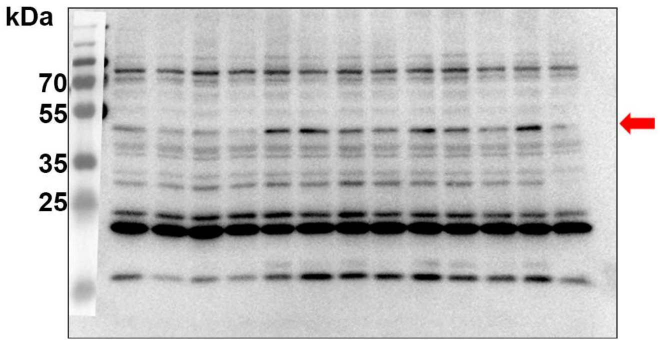

Full unedited Western Blot membrane incubated with anti-eEF1A1 (Abcam #ab118703)

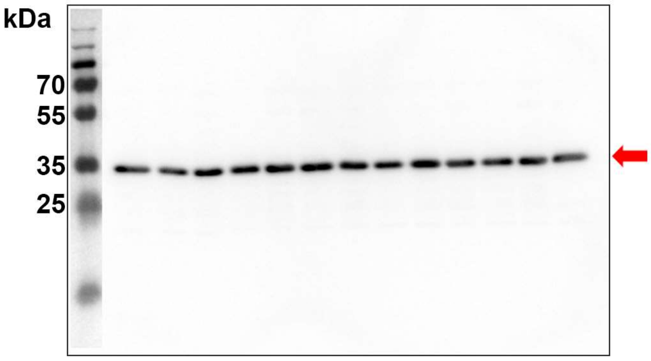

Full unedited Western Blot membrane stripped and incubated with anti-GAPDH (Fitzgerald #10R-G109a)

Source data to Figure 5E

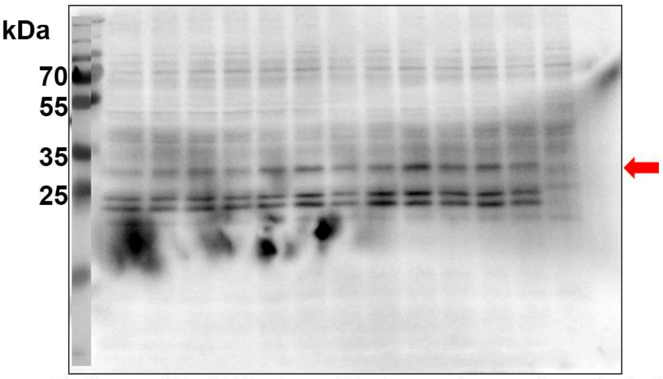

Full unedited Western Blot membrane incubated with anti-TIP30 (Abcam #ab177961)

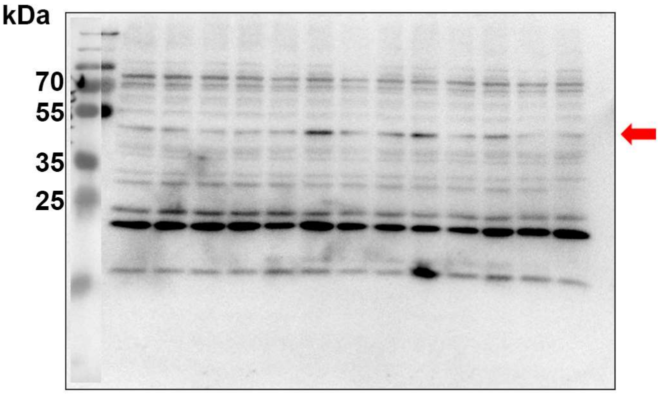

Full unedited Western Blot membrane incubated with anti-eEF1A1 (Abcam #ab118703)

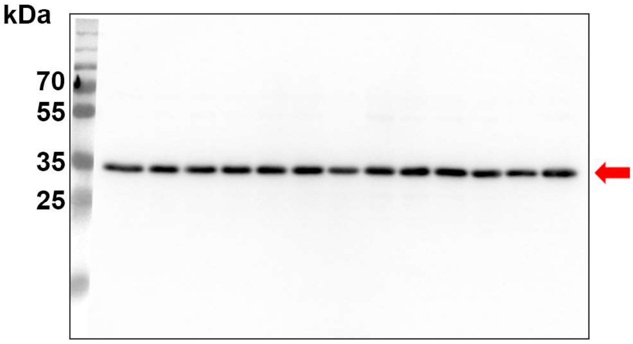

Full unedited Western Blot membrane stripped and incubated with anti-GAPDH (Fitzgerald #10R-G109a)

Source data to Figure 5G

shown in Fig. 5G

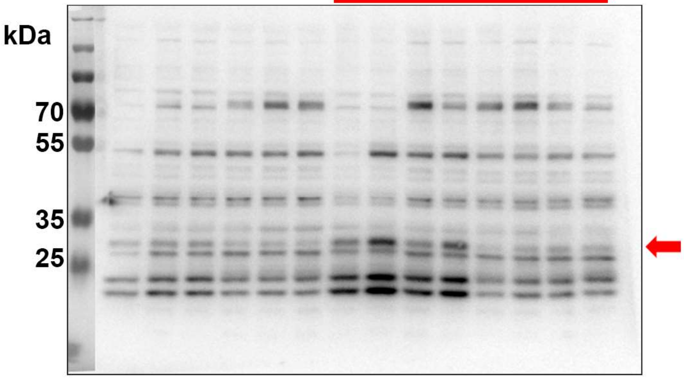

Full unedited Western Blot membrane incubated with anti-TIP30 (Abcam #ab177961)

shown in Fig. 5G

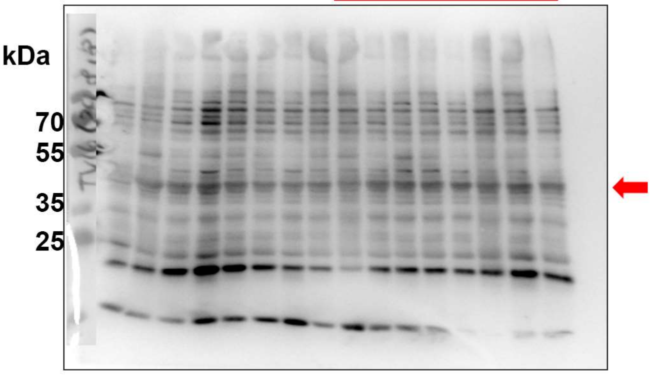

Full unedited Western Blot membrane incubated with anti-eEF1A1 (Abcam #ab118703)

shown in Fig. 5G

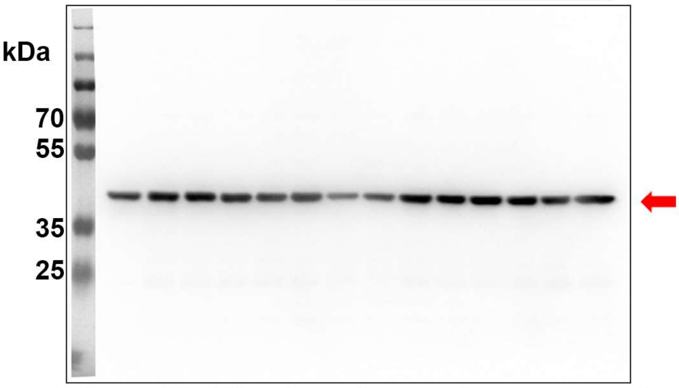

Full unedited Western Blot membrane stripped and incubated with anti-Actin (Sigma #2066)

Source data to Figure 5l

shown in Fig. 5l

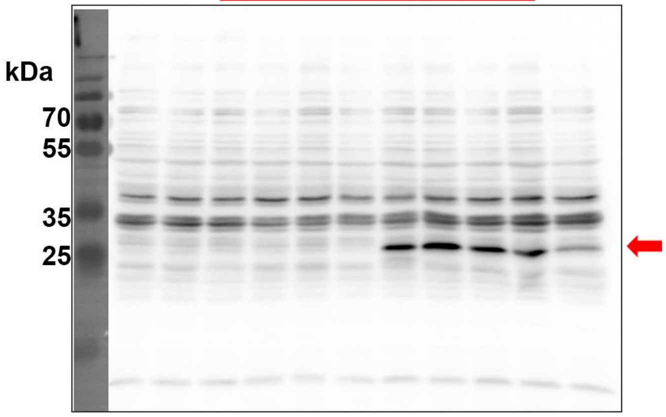

Full unedited Western Blot membrane incubated with anti-TIP30 (Abcam #ab177961)

shown in Fig. 5l

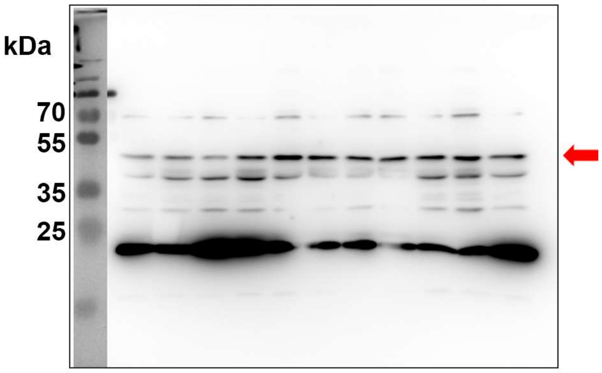

Full unedited Western Blot membrane incubated with anti-eEF1A1 (Abcam #ab118703)

shown in Fig. 5l

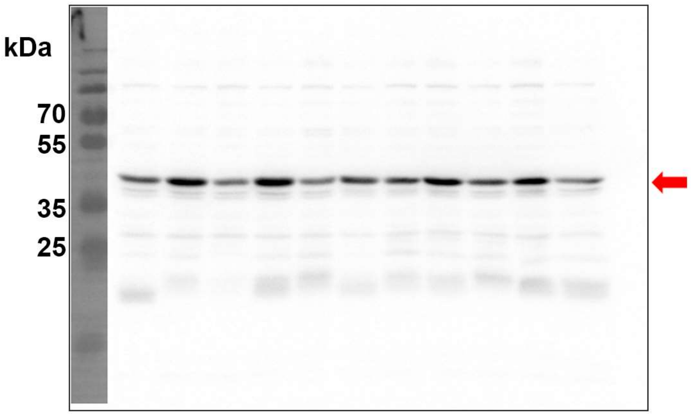

Full unedited Western Blot membrane stripped and incubated with anti-Actin (Sigma #2066)
