# Supplementary material for: TIP30 counteracts cardiac hypertrophy and failure by inhibiting translational elongation
Source: EMBO Mol Med. 2019 Aug 30;11(10):e10018. doi: 10.15252/emmm.201810018 (PMC6783653; doi:10.15252/emmm.201810018)
Supplement: Supplementary file 10 — Source Data for Figure 7 [file EMMM-11-e10018-s008.pdf]

Source data to Figure 7A

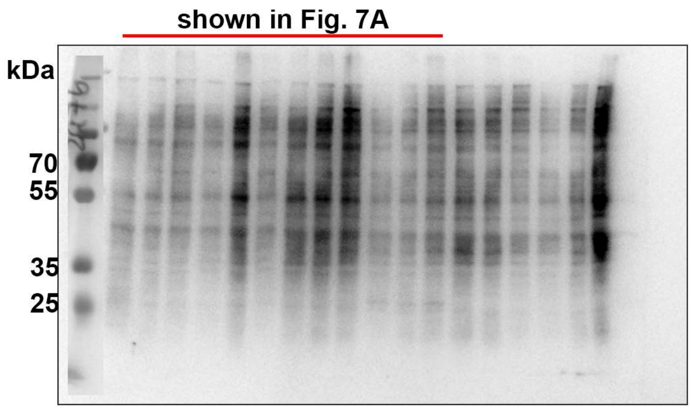

Full unedited Western Blot membrane incubated with anti-Puromycin (Sigma #MABE343)

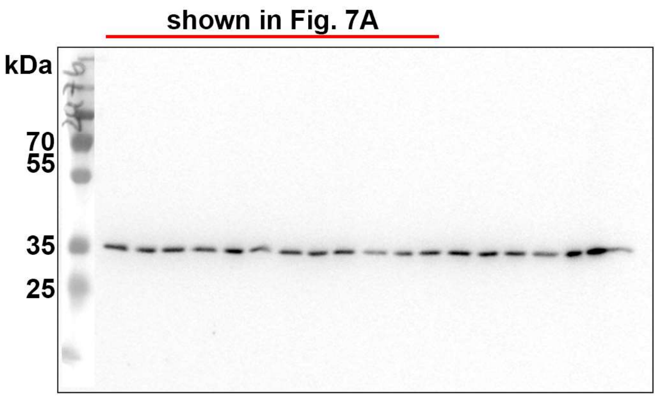

Full unedited Western Blot membrane stripped and incubated with anti-GAPDH (Fitzgerald #10R-G109a)

Source data to Figure 7I

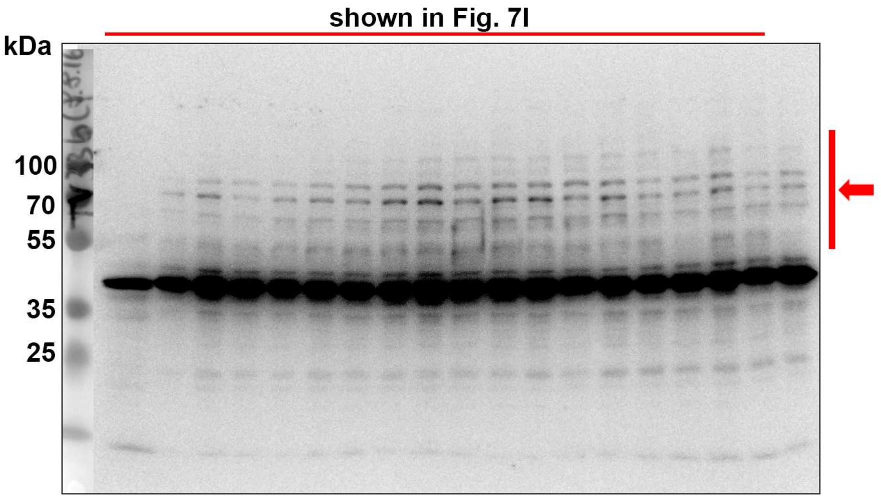

Full unedited Western Blot membrane incubated with anti-Puromycin (Sigma #MABE343)

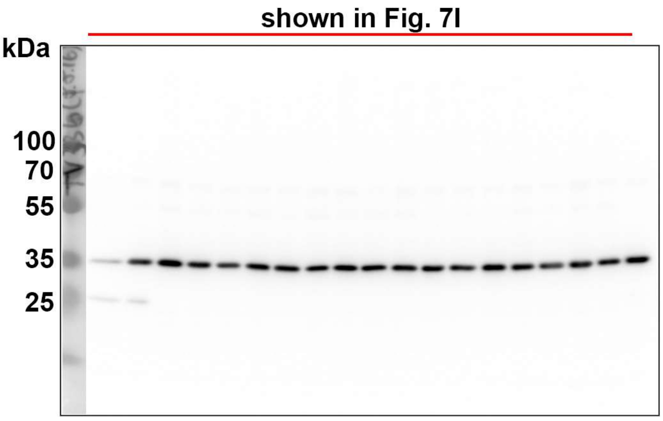

Full unedited Western Blot membrane stripped and incubated with anti-GAPDH (Fitzgerald #10R-G109a)
